# Socially interactive industrial robots: a PAD model of flow for emotional co-regulation

**DOI:** 10.3389/frobt.2024.1418677

**Published:** 2025-01-28

**Authors:** Fabrizio Nunnari, Dimitra Tsovaltzi, Matteo Lavit Nicora, Sebastian Beyrodt, Pooja Prajod, Lara Chehayeb, Ingrid Brdar, Antonella Delle Fave, Luca Negri, Elisabeth André, Patrick Gebhard, Matteo Malosio

**Affiliations:** ^1^ Affecting Computing Group, German Research Center for Artificial Intelligence (DFKI), Saarbrücken, Germany; ^2^ Industrial Engineering Department, University of Bologna, Bologna, Italy; ^3^ STIIMA, National Research Council of Italy, Lecco, Italy; ^4^ Human-Centered Artificial Intelligence, University of Augsburg, Augsburg, Germany; ^5^ Department of Psychology, University of Rijeka, Rijeka, Croatia; ^6^ Department of Pathophysiology and Transplantation, University of Milan, Milano, Italy

**Keywords:** human-robot interaction, socially interactive agents, affective computing, affect modeling, emotion (Co-)Regulation, social signals, pleasure, arousal

## Abstract

This article presents the development of a socially interactive industrial robot. An Avatar is used to embody a cobot for collaborative industrial assembly tasks. The embodied covatar (cobot plus its avatar) is introduced to support Flow experiences through co-regulation, interactive emotion regulation guidance. A real-time continuous emotional modeling method and an aligned transparent behavioral model, BASSF (Boredom, Anxiety, Self-efficacy, Self-compassion, Flow) is developed. The BASSF model anticipates and co-regulates counterproductive emotional experiences of operators working under stress with cobots on tedious industrial tasks. The targeted Flow experience is represented in the three-dimensional Pleasure, Arousal, and Dominance (PAD) space. We present how, despite their noisy nature, PAD signals can be used to drive the BASSF model with its theory-based interventions. The empirical results and analysis provides empirical support for the theoretically defined model, and clearly points to the need for data pre-filtering and per-user calibration. The proposed post-processing method helps quantify the parameters needed to control the frequency of intervention of the agent; still leaving the experimenter with a run-time adjustable global control of its sensitivity. A controlled empirical study (Study 1, N = 20), tested the model’s main theoretical assumptions about Flow, Dominance, Self-Efficacy, and boredom, to legitimate its implementation in this context. Participants worked on a task for an hour, assembling pieces in collaboration with the covatar. After the task, participants completed questionnaires on Flow, their affective experience, and Self-Efficacy, and they were interviewed to understand their emotions and regulation during the task. The results from Study 1 suggest that the Dominance dimension plays a vital role in task-related settings as it predicts the participants’ Self-Efficacy and Flow. However, the relationship between Flow, pleasure, and arousal requires further investigation. Qualitative interview analysis revealed that participants regulated negative emotions, like boredom, also without support, but some strategies could negatively impact wellbeing and productivity, which aligns with theory. Additional results from a first evaluation of the overall system (Study 2, N = 12) align with these findings and provide support for the use of socially interactive industrial robots to support wellbeing, job satisfaction, and involvement, while reducing unproductive emotional experiences and their regulation.

## 1 Introduction

The expanding market for collaborative robots (Cobots) (Knudsen and Kaivo-Oja, 2020) presents a novel opportunity to leverage Socially Interactive Agents (SIAs) to improve worker wellbeing in production line settings. SIAs could be employed to utilize *co-regulation* processes, where emotional regulation exhibited by one individual influences another’s (Järvelä et al., 2019). This approach has the potential to shift factory environments from a paradigm centered on efficiency towards a *value-driven era* (Schneiders and Papachristos, 2022) that prioritizes worker wellbeing and participation, aligning with the tenets of the “fifth industrial revolution” (Xu et al., 2021). By functioning as social companions embodied within the physical workspace of the production line, SIAs could mitigate negative experiences, particularly those associated with robotic manipulators and increased production demands.

Virtual SIA could act as mediators between cobots and their operators, promoting a lifelike social experience. SIA can move in human-like ways with sets of actions impossible for today’s industrial robots, while presence and physical embodiment may enhance the salience and perceived importance of lifelike interactions, compared to interactions with two-dimensional entities (Kawamichi et al., 2005).

However, the efficacy of SIAs hinges upon the development of an Avatar-Cobot behavioral model capable of anticipating and counteracting negative emotional states. Research by Nicora et al. (2023) explored how integrating a robot’s physical capabilities with an SIA’s verbal and non-verbal skills impacts user perception of the system as a social entity. The combination of both is promising.

This paper describes some of the achievements of the European project MindBot (Nicora et al., 2021) in applying a combination of SIAs and cobots in an industrial working scenario. The **main contribution** of this work is the description of a model and strategies to measure the PAD social signals (Pleasure, Arousal, Dominance) of a worker, and apply them for piloting his/her interaction with a pair of agents formed by a *cobot* (industrial collaborative robot) and an *avatar*, whose behavior merges into a single *covatar*.

**FIGURE 1:**
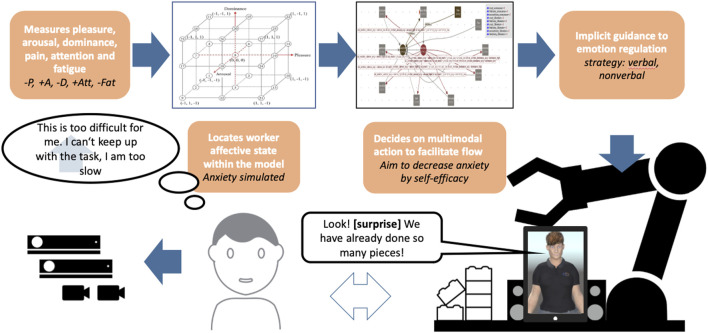
Abstract view of the application setup.

The top-level interaction cycle of a working cell is shown in Figure 1, where a worker collaborates with a cobot to perform a cyclical assembly task. The face and body motion of the worker are analyzed by a selection of AI modules extracting information about his/her PAD values, pain, and fatigue. The development of such a complex architecture faced **two main challenges**.

The first challenge relates to the explainability and control of system interventions as a reaction to workers’ experience. Research focusing on design principles for work cells using Cobots that consider operator mental-health (Nicora et al., 2021). We aim to establish theoretically grounded and transparent principles for facilitating social interaction through the integration of Socially Interactive Agents (SIAs) with robots, particularly collaborative robots/Cobots, in industrial settings. A systematic testing approach necessitates the ability to isolate and empirically evaluate individual components of the human-agent interaction. However, the inherent complexity of end-to-end systems precludes such granular analysis. Furthermore, existing research lacks a focus on dissecting these interaction aspects specifically for non-humanoid robots commonly employed in industrial environments. Therefore, this study represents a pioneering effort to address this critical gap in the current knowledge base.

Building on this foundation, the primary objective of this investigation is to develop and evaluate a transparent, theory-driven method for real-time emotional modeling. The method will focus on five key emotional states: boredom, anxiety, self-efficacy, self-compassion, and flow, collectively referred to as the BASSF model. In essence, BASSF aims to provide socially responsive interactions that promote self-efficacy and flow experiences, thereby enhancing worker wellbeing within production line settings (Nunnari et al., 2023). Within this framework, we delve deeper to empirically examine the specific relationship between Dominance and both Self-Efficacy and Flow.

The second main challenge of the development concerns the interpretation of human working experience from digital sensors.

While recent breakthroughs in AI (especially neural machine learning) have made automatic activity recognition much more precise, there are limitations to consider. For instance, research suggests that AI can estimate emotions from facial expressions with potential for real-world applications in fields that deal with human affect (Toisoul et al., 2021a). However, this holds true primarily in controlled settings. In real-time interactive situations, AI models often struggle due to unexpected factors. These include changes in lighting, camera quality, background noise, and user behavior that deviates from expectations. Simple models relying on pre-programmed thresholds and emotional triggers prove unreliable, leading to unrealistic responses from AI agents. Facial analysis data itself presents challenges. Raw PAD (valence, arousal, dominance) signals can be erratic, with sudden spikes and gaps. The data may not cover the entire predicted range [0-1], and its distribution is uneven and non-normal. Additionally, data streams can be interrupted when users move out of frame, turn away, or when the image quality is compromised by blur or distance.

There’s a concerning trend in research papers: crucial details are sometimes dismissed as mere “technicalities” and left out in favor of broader theoretical discussions. These details, often referred to as the “tricks” developers use to make their systems function, are often critical for replicating the research and verifying its findings.

In summary, this article provides two main contributions, addressing the two above-described problems: i) it describes in detail the BASSF explainable model of worker’s experience and the results of its validation, and ii) it describes strategies for post-processing PAD signals in order to drive a believable activation of the cobot + avatar interventions. The article also provides a first evaluation of the overall system which helps to situate the results of the BASSF evaluation within the goals of the system and understand them in the context of enhancing industrial robots for worker wellbeing.

The remainder of the paper is structured as follows. Section 2 presents state of the art work in social signal interpretation for interactive robot and avatar interaction and on the regulation of flow. Section 3 described the application domain and overall technical setup. Section 4 presents the development of the BASSF model and the definition of corresponding co-regulating interventions. Section 5 presents the method to map raw Pleasure, Arousal, and Dominance signals into the activation of cobot and avatar interventions. The main theoretical assumptions underlying the BASSF model are validated in Section 6. Section 7 presents a first evaluation of the overall system. Finally, Sections 8, 9 summarize our proposal and presents final observations.

## 2 Background and related work

In this section, we present work related to the creation of a system able to interpret social signals and model people’s emotional reactions. We first focus on the description of the PAD (Pleasure, Arousal, Dominance) social signals, following with a survey on the strategies to elaborate them. Then, we present work related to the interpretation of such signals to extract explainable cues on people’s emotion, and conclude with a few references on the control of avatars and cobots behavior in hybrid robot/avatar-human working environments. The last subsection highlights the main novelty of this work with respect to the related work hereby presented.

### 2.1 Modeling emotions with pleasure, arousal and dominance (PAD)

The PAD model postulates that all emotional experiences can be represented and differentiated using a three-dimensional framework (Pleasure - Displeasure, degree of arousal, and Dominance - Submissiveness), with values ranging from −1 to 1 on each dimension (Russell and Mehrabian, 1977; Mehrabian and Russell, 1974; Mehrabian, 1995). The dimensions are split in positive and negative values, like + P and -P for pleasant and unpleasant states Mehrabian (1996b) (Table 1). This divides the PAD Space into 8 octants, known as Octant Space or Eight States Model (Cao et al., 2008).

**TABLE 1:** The eight octants of the BASSF interventions space.

Activation code	Mehrabian (1996a)	Gilroy et al. (2009)	This work
+P+A+D	Exuberant	Flow	Flow
+P+A-D	Dependent	Impressed	Awe
+P-A+D	Relaxed	Relaxed	Relaxed
+P-A-D	Docile	Hopeful	Hopeful
-P+A+D	Hostile	Hostile	Hostile
-P+A-D	Anxious	Anxious	Anxious
-P-A+D	Disdainful	Disdainful/Dismissive	U-Boredom
-P-A-D	Boredom	Apathy	O-Boredom

Despite its widespread application across various disciplines Bakker et al. (2014), the PAD model has been criticised with regard to the Dominance dimension. Some researchers question its conceptual clarity of Dominance (Bakker et al., 2014).

Within the context of Flow, Gilroy et al. (2009) proposed a representation using the PAD model. This framework positions pleasure as a key predictor of Flow. Arousal represents the level of stimulation experienced during a task or interaction with an interface. Dominance relates to the skill-to-challenge ratio and is typically associated with feelings of control and influence. A situation where an individual lacks the necessary skills for a task would likely induce a submissive state (-D). However, challenging tasks can also be stimulating, potentially leading to a positive arousal state. Consequently, Flow is depicted as existing within the PAD space as a pleasurable (+P), aroused (+A), and dominant (+D) state, situated within the octant characterized by these positive values.

### 2.2 Social signals processing

Research on using social signals (like PAD) for robot and avatar interaction often lacks crucial details on how this information is actually used. A recent handbook on the topic (Lugrin et al., 2022) provides tools for processing social signals (Chapter 20.6), but neglects to explain how to “clean up” these signals for reliable application in driving agent behavior. While some tools offer “confidence” indicators, their role in improving system reliability remains unclear. The interpretation of this metric seems left to the specific needs of each development team, hindering overall understanding and consistency.

There are, however, some cases where low confidence detection is directly integrated into the behavior model. Carletto, for instance, is an agent that guides museum visitors through multiple rooms (Damiano et al., 2008). It infers the visitor’s location and if the signal is unclear, proactively addresses them with an informative message (“I cannot see you well. Can you move to the center of the room?”). Similarly, popular voice-activated smart home assistants like Alexa, Siri, and Google Assistant, despite experiencing high rates of false detections (Kurz et al., 2021), mitigate user frustration by directly acknowledging their inability to understand the user’s intent [2]. However, this approach is not universally applicable.

Dai et al. (2019) document the applications of pain detection in robot-assisted rehabilitation. This study explored the use of a humanoid robot to recognize pain in patients through facial analysis. While their models achieved high accuracy in detecting pain on individual, isolated images, real-time application required additional processing. They implemented a post-processing filter that analyzed a majority vote within a buffer of N consecutive frames (with N being greater than M, the number of votes needed for a pain flag). This filter helped account for inconsistencies in the real-time data stream. The study also highlighted the challenge of missing frames and the need for subject-specific optimization of the post-processing parameters.

EmmA, an agent designed by Gebhard et al. (2019), assists therapists in treating burnout patients. It analyzes user behavior through a mobile phone’s face camera, microphone, and other sensors. The authors acknowledge the importance of recalibrating models for individual users, but have not published further results on this aspect yet.

Arora et al. (2022) introduced Gloria, a socially interactive agent that supports patients during rehabilitation exercises. Gloria uses real-time analysis of facial expressions, eye gaze, and heart rate through a neural network to estimate the patient’s attentiveness, stress, and pain levels. However, due to the instability of these signals, Gloria does not react instantaneously. Instead, it analyzes the patient’s behavior over the past few minutes, triggering the avatar’s intervention at specific points within the therapy cycle. Our application adopts a similar “checkpoint” mechanism for improved reliability.

### 2.3 Flow, emotion (co-)regulation

Csikszentmihalyi (1975) described Flow as a highly engaging and intrinsically rewarding state characterized by enjoyment and effectiveness. This state is underpinned by six key components: Merging of action and awareness, centering of attention, loss of self-consciousness, feeling of control, coherent non-contradictory demands, and autoletic nature.

Beyond the core components, Flow is further influenced by the skill-to-challenge ratio, which is arguably the most critical aspect with regard to Dominance, as it directly impacts feelings of control (Massimini et al., 1987; Csikszentmihalyi, 1990). A balanced skill-to-challenge ratio is believed to foster Flow experiences, while a mismatch can lead to negative emotional states, for instance, boredom or apathy, stress, anxiety, or shame. To effectively respond to workers‘ emotional experiences of boredom and anxiety in a personally relevant manner, a deeper understanding of boredom itself is crucial. Boredom may exhibit diverse origins and functions and its multifaceted character needs to be adequately represented.

Within the production line context, boredom can manifest in two distinct ways beyond the common perception of under-stimulation. The first type arises from over-challenge, potentially due to time constraints or competitiveness among workers. Alternatively, over-challenge boredom can be triggered by organizational-level failures. A second form of over-challenge boredom, related to task-focus, can emerge from repetitive and seemingly meaningless tasks, characteristic of many assembly line environments (Feuchter and Preckel, 2022). In contrast, self-focused boredom arises when individuals become preoccupied, dissatisfied, or frustrated with themselves. In that case, self-focused boredom serves as a defense mechanism to prohibit chronic high-stress states (Feuchter and Preckel, 2022). Individuals may subconsciously enter a state of boredom to mitigate the experience of negative and self-threatening emotions (Nathanson, 1994). This mechanism is depicted in the known connection between over-challenge boredom, anxiety, and identity threats (Csikszentmihalyi, 1990; Feuchter and Preckel, 2022).

Negative emotions directed inwards (self-referential emotions) can intensify self-focused attention, potentially leading to a state of boredom (Bambrah et al., 2023). Furthermore, limited self-awareness may lead to boredom, to avoid confronting potentially negative emotions. Interestingly, there is a correlation between over-challenge boredom and diminished Self-Efficacy when considering self-regulation and achievement. However, the same association is not encountered with under-challenge boredom (Tze et al., 2014).

Effective emotional regulation of negative emotions plays a critical role in facilitating Flow experiences, as it can remove obstacles that may impede Flow and can influence the perceived skill-to-challenge ratio. Cognitive reappraisal, for instance, allows individuals to reinterpret the demands of a situation, potentially altering their appraisal of the skill-to-challenge balance (McRae and Gross, 2020).

Emotion co-regulation offers a framework for providing guidance on emotional regulation strategies (Gergely, 2004; Järvelä et al., 2019). However, the effectiveness of this approach is contingent on contextual factors, the individual’s specific emotional state, and inherent individual differences. Implicit guidance is less disruptive to the user’s experience (Heimbuch and Bodemer, 2017) and may be appropriate in situation requiring task-focus. Conversely, explicit guidance, which may involve direct prompts for action, has the potential to interrupt ongoing tasks (Loksa et al., 2016). While real-world scenarios may involve a blend of both implicit and explicit elements, this distinction is valuable for informing design decisions within specific application contexts, such as production line settings where maintaining focus on the task at hand is paramount. The representation of such strategies in the system allows personalising the co-regulation (Gergely, 2004; Järvelä et al., 2019) to help the worker regulate themselves and even possibly appropriate new regulation strategies. In order to achieve co-regulation, the regulating partner needs to partake in the emotional experience of the other, making themselves and the other aware of the emotional experiences, containing it (Fonagy et al., 2018), empathizing, and regulating if needed to support the other’s emotion regulation and emotional development (Fonagy et al., 2007). In the current context, this amounts to being able to monitor and dynamically respond to the worker’s emotional experiences. Such co-regulation processes have been researched in task-related co-regulation before, especially in the context of collaborative group work (e.g., Järvelä et al., 2019).

### 2.4 Cobot/avatar behavior

Virtual agents that provide emotional support specifically with regard to Flow are limited but provide optimistic results. D’Mello and Graesser (2013) presented AutoTutor and Affective AutoTutor, two intelligent tutoring systems trace emotional states to increase engagement and Flow for learning. Pagalyte et al. (2020) introduce the idea of incorporating Dynamic Difficulty Adjustment (DDA) through the use of Reinforcement Learning (RL) in turn-based battle video games to induce game Flow. Samrose et al. (2020) investigated the potential of an empathetic conversational agent to alleviate boredom. These test different interaction settings and deal with virtual agent behavior, but do not explicitly make use of emotion co-regulation in a systematic way. Opportunities for emotion co-regulation arise when combining avatars with cobots who co-habit and work in the physical space of the production line (Lugrin et al., 2022).

Analysis of human-human interaction highlights the importance of verbal and non-verbal communication. In every socially interactive scenario, motor correlates such as lip-syncing, head nods, deictic gestures, and gaze movements are abundant and play a great role in expressing emotions, intentions and establishing common ground in communication (Mavridis, 2015). However, such capabilities are missing in current industrial cobot installations, increasing the risk for social isolation of their operators. There is scarce knowledge regarding how industrial robots can be adapted to improve the emotional experience and reduce health risks. There is evidence that people project themselves onto non-humanoid robotic devices (Aymerich-Franch et al., 2017), and preliminary studies (Nicora et al., 2023) are trying to understand how humans perceive them and what roles they ascribe to them.

Research in manufacturing has established that a cobot’s speed and acceleration significantly influence the experience of workers collaborating directly with it (Ojstersek et al., 2023). Studies by Kato et al. (2010) and Arai et al. (2010) demonstrate that worker stress levels decrease as cobot speed is reduced or the distance between cobot and operator increases. Following this guideline, in our work, we modulate the robot speed to promote flow.

### 2.5 Main innovation points

With respect to the surveyed literature, this work presents four main novelties.

First, our setting comprises three elements: (i) A collaborative robot (cobot), which is (ii) embodied by an accompanying avatar for (iii) the industrial manufacturing domain. None of the surveyed work presents this combination of features all together.

Second, this is the only work known to us that uses the Pleasure, Arousal, and Dominance combination to interpret social signals and use them as input for the model that infers humans subjective experience.

Third, we propose a novel model (BASSF, Section 4) that classifies the experience of workers and defines theory-based interventions of the Covatar behavior to promote workers’ flow. The model relies on the explicit interpretation of the PAD signals of the workers, and, with respect to many current models based on neural black boxes, it is based on transparent rules following on psychological theories. This approach renders the model explainable, which allows testing and making informed adjustments. We have demonstrated here how the model can be tested by scrutinizing the assumptions of the PAD representation of the subjective user experiences.

Fourth, many of the surveyed works use several kinds of “social signal” in the broad sense–i.e., of any measurable temporal signal which can help inferring users’ experience–like body motion, facial expressions, heart rate, skin inductance, and the like. However, very few details are provided on how to carefully filter and clean such signals. In this work, we present a careful description of the PAD signals, performing statistical analyses, and provide a method to post-process and calibrate such data in order to make them useable in real-world settings; for example, to better generalize among different users and reduce the effect of environmental noises (Section 5).

## 3 Application overview

This section describes separately all of the hardware and software components involved in the realization of the application already depicted in Figure 1.

### 3.1 System setup

**FIGURE 2:**
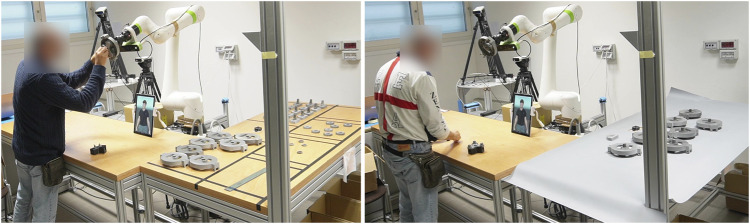
Photos of the working space. The operator stands in front of his desk, where he joins his parts with the ones given by the cobot. The desk on his right is the operating space where the cobot picks his components. The tablet showing the avatar stands on the operator desk, just in front of the cobot rotating base. Left: phase 1 setup - The robot and the operator have completed their subassembly and the collaborative joining is ongoing. Right: phase 2 setup - The robot has brought the subassembly towards the operator, who is still working on his parts.

To simulate an industrial workcell, we built a replica in a laboratory environment (see Figure 2). The setup features two L-shaped tables creating separate workspaces for the operator and the cobot. A Fanuc CRX10iA/L collaborative robot (cobot) is positioned in front of the operator, along with a tablet displaying the virtual agent (avatar). The system incorporates a front-facing HD camera (described in Section 3.3) to analyze the user’s social signals, including Ekman facial expressions, pleasure, and arousal. Additionally, the face camera signal is used to estimate the level of *pain* of the worker [using the method described by Prajod et al. (2022a)], and two Kinect cameras used within a separate system to estimate user *fatigue*, as detailed by Brambilla et al. (2022). These two last variables are part of the global system, but unused by the emotion co-regulation approach presented in this paper.

The entire setup is controlled by four interconnected machines using the ROS framework (Quigley et al., 2009): (i) a Linux PC that manages all robot control functions; (ii) a Windows PC that executes the Social Signals Interpretation (SSI) module and the Visual Scene Maker (VSM) program, which controls the covatar behaviors; (iii) a Windows PC that analyzes data from the Kinect sensors to estimate user fatigue; and (iv) a Windows Tablet that runs the avatar Unity application.

### 3.2 Assembly task

The study focuses on a collaborative product assembly task in an industrial setting. The robot handles assembling specific components, while the human operator takes care of others. The final step involves them working together to join the two sub-assemblies, as described by Mondellini et al. (2023). A description of the assembly components is available at https://doi.org/10.5281/zenodo.5675810.

One *assembly cycle* involves the robot putting together its assigned parts before presenting the completed sub-assembly to the operator. Our reference assembly task, which simulates realistic conditions, lasts about 50 s when setting the speed of the Robot to the maximum allowed by EU safety regulations. However, the corresponding assembly task performed simultaneously by the worker can be finished in about half of that time; thus giving the operator significant idle time. Workers’ goal was to complete as many assemblies as possible, but they had to wait for the Cobot to be ready before starting a new assembly. Hence, the extended waiting period could lead to boredom and frustration over time.

In order to investigate the opposite effect, when the robot finishes first, also realistic, which could result in stress for workers, we configured also a faster, reduced version of the robot assembly. We tested both possibilities and their effect on flow in Study 1 (Section 6), while the execution of the reference “slow speed” task in conjunction with the intervention of the avatar is the subject of Study 2 (Section 7).

### 3.3 Facial emotion detection

We deployed an SSI (Wagner et al., 2013) pipeline to capture a view of the worker using the front camera, identify and crop the face [using MediaPipe (Bazarevsky et al., 2019)], and predict: i) 7 discrete classes (Neutral, Happy, Sad, Surprise, Fear, Disgust, Anger) and ii) two continuous values (Valence and Arousal). The facial expressions of the participants were captured using a front camera (Logitech C920 Pro HD). The face-cropped images served as input to the deep learning model trained using TensorFlow on NVIDIA GeForce GTX 1060 6 GB GPU.

The model was trained on the AffectNet dataset (Mollahosseini et al., 2019), which was cleaned following the procedure mentioned in Toisoul et al. (2021a). This step yields 218,827 images from 7 emotion classes, split into 85% for training and 15% for validation. We used the images indicated in Toisoul et al. (2021b) as the test set (2,457 images).

The system employed a pre-trained VGG16 model, originally developed by Simonyan and Zisserman (2014). This model was trained on the massive ImageNet image database (Russakovsky et al., 2015). We then fine-tuned the entire VGG16 network using the specific training data they collected for this project. Following the VGG16 network, we added a fully connected layer. This layer then connects to three separate prediction layers. The first layer predicts one of seven emotions (using Softmax activation). The other two layers predict the valence (using Tanh activation) and arousal (using Tanh activation) levels.

All input images were scaled to the default VGG16 dimensions (224 × 224) and were fed to the model in batches of 16. To introduce more variances in training images, we used data augmentation options provided by TensorFlow: width shift (range = 10%), height shift (range = 10%), zoom (range = 10%), and horizontal flip. We used the SGD optimizer with an initial learning rate of 0.001 and reduced it by a factor of 0.1 after 70,000 steps. Similar to Prajod et al. (2022a); Prajod et al. (2022b), we use the focal loss function for the discrete emotion classification. For valence and arousal, we used the shake-shake loss function (Toisoul et al., 2021b), which is a combination of CCC (Concordance Correlation Coefficient), PCC (Pearson Correlation Coefficient), and MSE (Mean Squared Error) losses. We employed the early-stopping mechanism (patience = 5) to prevent over-fitting of the model, i.e., we stopped training the model when the total loss on the validation set did not decrease for 5 consecutive epochs.

The system achieved a success rate of 76% on the test set, based on both accuracy and F1-score, for the task of classifying distinct emotions. This performance aligns with prior research in this area (Mollahosseini et al., 2019; Toisoul et al., 2021b). To assess the model’s ability to predict valence and arousal levels, we adopted the same metrics used in similar studies: Concordance Correlation Coefficient (CCC), Root Mean Squared Error (RMSE), and Sign Agreement (SAGR). Our model’s performance on these metrics was comparable to the best existing systems, achieving a CCC of 0.852, RMSE of 0.266, and SAGR of 83.1% for valence prediction, and a CCC of 0.763, RMSE of 0.277, and SAGR of 81.2% for arousal prediction.

### 3.4 Dominance estimation

The system does not directly output dominance as a standalone value. Instead, it infers dominance by combining the strengths of the six Ekman facial expressions in a linear fashion.

This approach relies on a mapping established by Mehrabian (1995), who assigned specific PAD values to various emotions. We determine the dominance level associated with each Ekman expression using the following coefficients: neutral (0.0), surprise (−0.16), happiness (0.46), sadness (−0.33), disgust (−0.36), anger (0.25), and fear (−0.43). In real-time, the system calculates the user’s dominance level based on a linear combination of these weighted coefficients applied to the actual strengths (softmax distribution) of the detected Ekman expressions. More formally:

$$D=\underset{e}{\sum }p_{e}*c_{e},\text{with}\underset{e}{\sum }p_{e}=1$$

Where e cycles through all the six Ekman expressions (plus neutral), $p_{e}$ is the value of the softmax output for the emotion, and $c_{e}$ is the coefficient described before (plus $c_{\mathrm{neutral}}=0.0$ ).

### 3.5 Cobot control

The software architecture prioritizes expandability and hardware independence. To achieve this, the system utilizes the Robotic Operative System (ROS) framework (Quigley et al., 2009). This, combined with the modular design (described below), allows for deployment on various robotic cells. This flexibility makes the system adaptable to a broad range of collaborative robotics applications. The robot controller transmits information through ROS. This information includes the robot’s current control state and mode (updated at 1 Hz) and its position, velocity, and acceleration (updated at a faster rate of 15 Hz). Additionally, the controller offers various services that the behavior management software can leverage to control the robot and execute the assembly task. The main high-level commands are:

• set_tcp_target - moves the gripper in a given position and orientation.
• set_gripper_action - open/closes the gripper.
• set_detection - activates the on-gripper mounted camera to find the required assembly piece.
• set_max_tcp_velocity - imposes a maximum movement speed to the gripper.
• set_max_tcp_acceleration - imposes a maximum acceleration to the gripper.

### 3.6 Avatar design, deployment and features

**FIGURE 3:**
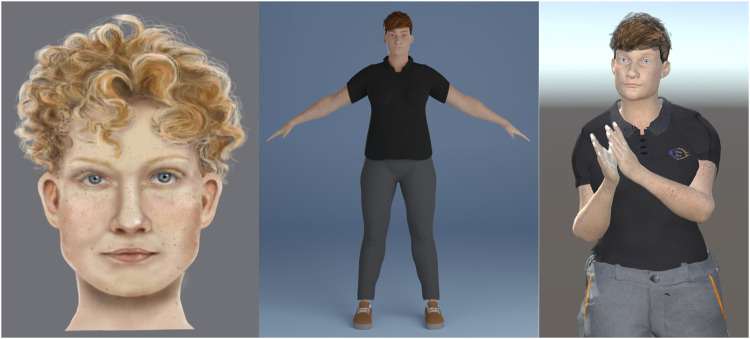
Avatar design and implementation. Left: a sketch for the design of the face. Center: an early off-line rendering of the character in Blender. Right: A screenshot of the real-time rendering in Unity.

Figure 3 showcases concept art and screenshots of the cobot’s virtual companion, the avatar. A graphic artist created the avatar, drawing inspiration from well-known actresses and models. The design prioritized two key aspects:

1. Androgynous Appearance: The avatar avoids stereotypical features of beauty and maintains a gender-neutral look to minimize potential gender bias.
2. Work Environment Integration: The avatar is clad in work clothes to blend seamlessly with the industrial setting and foster a sense of connection with the human worker.

The artist used Blender 3D software (https://www.blender.org) for character creation, aided by the MB-Lab character generation plugin (https://github.com/animate1978/MB-Lab). To enable real-time rendering and integration within a Unity application, the character underwent further editing and adaptation using the YALLAH framework (https://github.com/yallah-team/YALLAH) (Nunnari and Heloir, 2019).

The Unity application runs on a portable Windows tablet and establishes a Websocket network server that receives commands from remote controllers. These commands allow the avatar to perform various actions: speak out a given text message, look around the environment, rotate its body, play pre-recorded animation sequences, adjust the camera view distance.

### 3.7 Cobot and avatar behavior

The Visual Scene Maker (VSM) tool (http://scenemaker.dfki.de) (Gebhard et al., 2012b) was used to design the overall interaction between the cobot and the avatar. VSM’s user-friendly interface allows non-programmers to visually configure the behavior logic for these collaborative agents.

**FIGURE 4:**
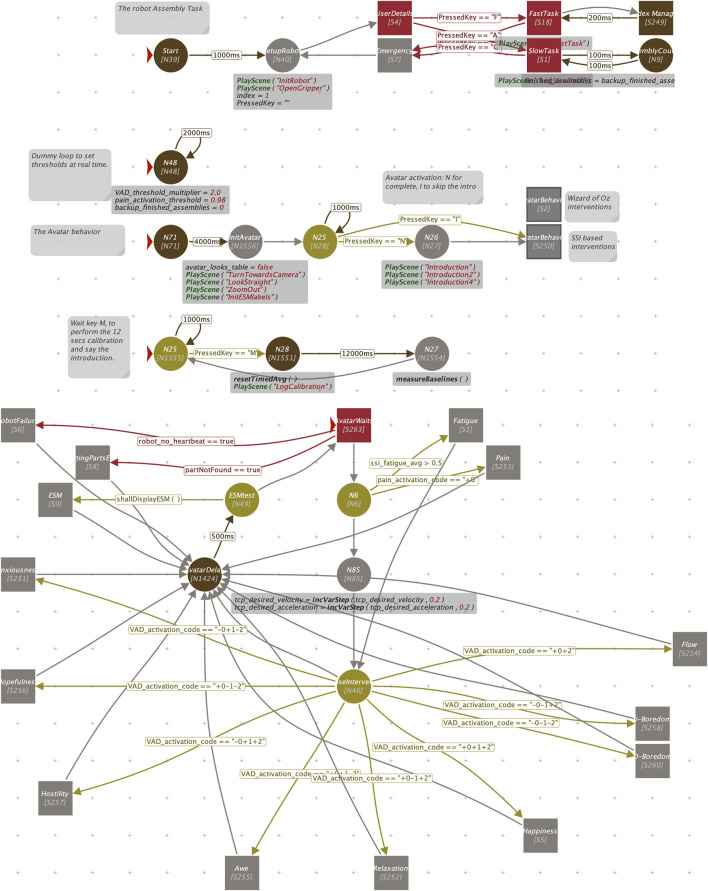
Top: the top-level of the VSM project driving Cobot and Avatar behavior. Bottom: a view of the sub-node implementing the decision strategy of the BASSF model.

The top of Figure 4 depicts a high-level view of the VSM scene flow managing the entire project. It consists of three primary subprojects: top, Controls the cobot’s behavior; Center, Manages the avatar’s behavior; Bottom, Calibrates the user’s facial expression recognition. VSM is extended with a custom plugin that handles several tasks:

• Communication with SSI Module: Receives data on fatigue, PAD (pleasure/valence, arousal, dominance), and Ekman facial expressions.
• Cobot & Avatar Communication: Facilitates communication between the VSM system and both the cobot and avatar.
• Dominance Derivation: Calculates the dominance level based on the detected Ekman expressions.
• Activation Code Strategy: Implements the strategy (described in a following section) to generate an “activation code” from the PAD data streams.

The bottom part of Figure 4 shows the main node implementing the BASSF activation strategy. The squared red node at the top center is the entry point. It performs the main tests to check for hardware errors (missing parts on the robot table, or communication failure). The following node (N6) is an explicit test on detected pain or fatigue (not covered in this paper). Then, after a small increase in the robot operation speed (velocity and acceleration), the yellow node at the center acts as dispatching center: according to the value of the activation code, it activates the node implementing the suitable activation strategy. For example, code -0-1+2 (corresponding to -P-A+D) triggers the activation of the U-Boredom node. All of the activation nodes converge then into the execution of a delay node that connect back to the starting node, thus closing the avatar behavior waiting loop.

## 4 The BASSF/PAD co-regulation model

The BASSF model co-regulation model allows making decisions on personalised situation-based feedback to support flow. It is implemented in VSM and is the controller of the cobot and avatar behavior in combination (Figures 1, 4). It is designed to track the worker’s affective experiences continuously. For this, the PAD model of affect by Mehrabian and Russell (1974), Cao et al. (2008), Gilroy et al. (2009) is used as data source. Their model uses three dimensions (pleasure - displeasure; aroused - unaroused; dominant - submissive) to differentiate between every possible affect. These three dimensions can be visualized as a three-dimensional space that is further subdivided at each axis. Those eight subspaces (Octants) can be labeled by their axis and their sign (Table 1). Then, as part of the BASSF model definition, from the PAD octants appropriate interventions are selected through a decision algorithm to assist. The interventions realise the co-regulation with the participant and support Flow experiences.

This research aims to improve the transparency, user-adaptability, and testability of the BASSF model by exploring the differential causes of boredom and its connections to stress and anxiety, common workplace emotions that can also influence flow (Massimini et al., 1987). The experiment focused on inducing prolonged stress, which can potentially be regulated through boredom (Feuchter and Preckel, 2022; Nathanson, 1994). We operationalized prolonged stress caused by overwhelming time pressure as a state of anxiety within the PAD space, characterized by negative pleasure (-P), high arousal (+A), and low dominance (-D). We then hypothesize that anxiety, an experience associated with low dominance, might eventually lead to boredom that is also regulated by low dominance. Building on Gilroy et al. (2009) definition of boredom as -P, -A, -D, we define the entire low-pleasure, low-arousal region of the PAD space as “regulated, overwhelming boredom” (O-Boredom). To distinguish this from another type of boredom, we introduce the concept of “underwhelming boredom” (U-Boredom) (Fisher, 1987), associated with negative pleasure (-P), low arousal (-A), and high dominance (+D).

The BASSF model defines covatar responses for emotional states within the PAD space that are considered unfavorable for achieving Flow. These covatar reactions provide guidance on how to regulate emotions and are intended to co-regulate both boredom and anxiety experiences. Each octant of the PAD space, representing a specific emotional combination, has its own set of potential interventions. However, the system prioritizes the user’s self-regulation efforts. Interventions are only triggered if the user is not effectively regulating their emotions towards a more productive state. To avoid disrupting the user’s focus on work tasks, the covatar’s guidance is delivered subtly and implicitly.

This system aims to support workers in achieving a state of “flow.” Flow is a well-being-promoting state characterized by enjoyment, productivity, and a sense of being completely absorbed in the task at hand (Csikszentmihalyi, 1975; Csikszentmihalyi, 1990). A key factor influencing flow is the balance between challenge and skill level. When these two elements are mismatched, negative emotions like anxiety, stress, and boredom can arise. These emotions hinder flow if left unchecked. The BASSF model tackles this challenge through both direct and indirect interventions:

• Indirect Assistance:
  • Implicit Emotion Regulation Guidance: The model subtly guides the worker towards regulating their emotions, thereby reducing the impact of negativity (Heimbuch and Bodemer, 2017).
  • Error Prevention: By intervening when errors occur (from either the worker or the cobot), the model helps prevent negative emotions from arising in the first place.
• Direct Assistance:
  • Challenge-Skill Balance Adjustment: The model directly influences the worker’s perception of the challenge-skill balance. This can involve, for example, influencing the worker’s self-efficacy beliefs.
  • Cobot Work Rate Adaptation: The model can directly adjust the cobot’s work pace to better align with the worker’s capabilities.

The research team configured 16 different interventions based on a worker’s emotional state within the PAD space (pleasure, arousal, dominance). Table 2 showcases 3 examples of these pairings. When an intervention is triggered, the avatar (the cobot’s virtual companion) performs two actions: Verbal Feedback, the avatar turns towards the worker and delivers a pre-selected message; Cobot Speed Adjustment, the cobot’s speed and/or acceleration are modified based on the chosen intervention. It is important to note that for situations where multiple interventions are applicable, the system randomly selects one to execute.

**TABLE 2:** Examples for 3 of the 16 configured interventions.

Octant	Intervention	Avatar behavior	Cobot behavior	Theoretical justification
-P+A-D	Self-Efficacy against Anxiety	Surprised expression. Says: “Look at that! We have already done so many pieces!”	–	Focus attention on the shared achievement to increase Skill-to-Challenge Ratio and ease the pressure by reminding them that they are a team Lackas (2021)
-P-A+D	Self-Awareness against U-Boredom	Head tilted to the right. Bending hips. Says: “Are you okay over there? Let me know if you need anything!”	Increase acceleration and velocity	Increase Self-Conscious- ness and Task-Awareness to reduce boredom via socio-cognitive conflict and increased challenge, while remaining caring Chehayeb et al. (2021); Bambrah et al. (2023)
-P-A-D	Self-Compassion against O-Boredom	Moderate zoom-in. Short compassionate smile. Says: “You are doing great! Everybody would be stressed at this speed”	–	Increases Self-Compassion to facilitate self-regulation and cognitive reappraisal Lackas (2021)

## 5 Mapping PAD signals to interventions through the BASSF model

This section focuses on how the system translates the continuous PAD (pleasure, arousal, dominance) values coming from the facial expression analysis (SSI module) into a discrete BASSF activation code (refer to Table 1). This activation code ultimately determines whether a cobot/avatar intervention is triggered.

A critical aspect of designing this mapping was managing the frequency of avatar interventions. In other words, we needed to establish thresholds defining how significantly the PAD values must deviate from the center (neutral) state to activate an intervention. Setting thresholds that are too low would lead to excessive intervention, potentially becoming bothersome for the worker. Conversely, thresholds that are too high would render the avatar inactive, limiting its usefulness.

Our initial tests revealed that frequent interventions from the covatar could be distracting or even annoying for the worker. To minimize disruption and ensure the worker stays focused on the task at hand, we decided to restrict feedback to specific points in the assembly process - namely, after the completion of each sub-assembly. While the avatar remains visible throughout the shift as a companion, we aimed to limit interventions to around 5 over a typical 30-minute work session. This approach balances the covatar’s supportive presence with avoiding interruptions to the worker’s workflow.

### 5.1 Baseline data collection

To assess the system’s potential, we used the data recorded from the participants to Study 1 (described later in Section 6). For technical reasons, however, we were able to record the social signals for only 14 of the 20 participants. Each participant repeatedly performed the assembly task described in Section 3.2 over a 30-minute period. It is important to note that this initial phase was non-interactive: while we performed a calibration procedure to gather statistical data, the cobot and avatar did not respond dynamically to the workers’ emotions or actions. Instead, we pre-programmed the interventions to occur at specific points in time. This allowed us to collect preliminary feedback on how users perceived the presence of a virtual agent during the assembly task.

### 5.2 Initial tentative model

Our initial approach to activating interventions was based on a simple distance check from a central neutral zone. Imagine each PAD dimension (pleasure, arousal, dominance) as a spectrum with a threshold value set at 2.0. This threshold divides each dimension into three zones: low<0.3≤neutral≤0.7<high . An intervention is triggered only when all PAD signals fall outside the neutral zone simultaneously. This ensures the system does not intervene for minor emotional fluctuations.

Such a simplistic model was based on the following assumptions:

1. The PAD signals are continuous and accurate;
2. The PAD values span over the whole normalized range [0,1];
3. When a user has a neutral facial expression, then the P, A, and D values are at 0.5;
4. Over an experiment session, the PAD signals have a normal distribution; and
5. An intervention should be activated when the three PAD values *simultaneously* exceed the neutral zone.

Upon reviewing the raw PAD data plots, it became clear that our initial assumptions about emotional states and intervention timing were inaccurate. This resulted in interventions firing erratically and unevenly. In some cases, the avatar remained inactive for extended periods, while in others, it provided repetitive feedback. The following sections detail the strategies we implemented to create a more reliable and natural intervention model.

### 5.3 Signal pre-filtering

The system initially relied on raw PAD signals from the emotion prediction model. However, these signals suffered from two key limitations:

• Jitter and Spikes. The data exhibited significant fluctuations, particularly when a user’s face first entered the camera’s view.
• Missing Data. Due to factors like user movement or head rotation, the face recognition system frequently lost track of the user, resulting in gaps (holes) in the PAD data stream.

To address these issues, we implemented a two-pronged approach:

• Median Filtering: We filter the incoming data by calculating the median of the past 5 PAD samples (at a 5 Hz input frequency, this smoothes the data over the last second). This helps mitigate the impact of sudden spikes and data fluctuations.
• Time-Aware Buffer: We employ a buffer that discards any PAD values older than 1 s. This prevents the median calculation from being skewed by outdated data points when the user’s face reappears after a period of being out of view.

This combined filtering approach ensures the intervention system relies on a more stable and reliable representation of the user’s emotional state.

### 5.4 The need for calibration

**FIGURE 5:**
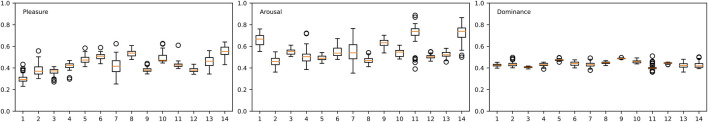
Boxplots comparing the calibration data among users.

We discovered a limitation in the model predicting pleasure, arousal, and the Ekman expressions used to infer dominance. This model does not have a universal “neutral” baseline. In other words, even when different participants stared at the camera with neutral expressions, the model produced statistically different results for each person (see Figure 5). To confirm this variation, we performed Kruskal–Wallis tests (Kruskal and Wallis, 1952) and found statistically significant differences in the medians for all three variables (pleasure, arousal, and dominance) across the 14 participants (*p*-value < 1E-90). This implies we cannot assume a single reference point for a neutral facial expression.

Therefore, the system requires calibration for each user. During calibration, the system establishes a baseline specific to that individual, allowing it to interpret subsequent emotional expressions relative to their unique starting point. In essence, assumptions about the user’s emotions are made in comparison to their own calibrated neutral state.

To address the need for user-specific baselines, we implemented a calibration process. At the start of each session, participants are instructed to record 12 s of neutral facial expression, characterized by relaxed muscles and closed lips. In more matematical terms, the system calculates the median values $\left(M_{P},M_{A},M_{D}\right)$ for pleasure, arousal, and dominance from each user’s calibration data. These medians then serve as individual baselines for interpreting subsequent emotional data acquired during the work session.

### 5.5 Overcoming data skewness

Our analysis revealed that the PAD signal distribution throughout a 30-minute work session deviates from a normal distribution and exhibits skewness.

This conclusion is supported by two findings. D’Agostino and Pearson’s normality tests (D’Agostino, 1971; D’Agostino and Pearson, 1973) were conducted for all 14 participants across the three PAD variables (pleasure, arousal, dominance). In each case, we were able to reject the null hypothesis that the data followed a normal distribution. Fisher-Pearson skewness coefficients were calculated for the data, indicating a strong skew in most cases. These results imply that a single, symmetrical threshold centered around the median value would not be effective.

To address this skewness, the system adopts a two-step approach:

• Centering. The data is first centered based on the individual’s calibration results (as described previously).
• Asymmetric Thresholds. Separate thresholds are then defined for positive and negative deviations from the centered values.

We calculated activation thresholds (denoted by T ) for each participant based on their calibration data. The process involved two steps. First, centering: the data points (samples) $S_{d}$ were first centered around the individual’s calibration means for pleasure, arousal, and dominance (d∈P,A,D) . Second, threshold calculation: for each centered data set $S_{d}^{\prime }$ , we split positive values $\left(S^{\prime +}\right)$ and negative values $\left(S^{\prime -}\right)$ :

$$S_{d}^{\prime }=S_{d}-M_{d}=S_{d}^{\prime +}\cup S_{d}^{\prime -}$$

$$\forall p\in S_{d}^{\prime +}\to p\geq 0$$

$$\forall p\in S_{d}^{\prime -}\to p<0$$

and we then calculated the root mean squared error (RMSE) separately for the positive values $\left(S^{\prime +}\right)$ and negative values $\left(S^{\prime -}\right)$ w.r.t. zero:

$$E_{d}^{+}=\sqrt{\frac{\sum p^{2}}{\left|S_{d}^{\prime +}\right|}},p\in S_{d}^{\prime +}$$

$$E_{d}^{-}=\sqrt{\frac{\sum p^{2}}{\left|S^{{\prime -}_{d}}\right|}},p\in S^{{\prime -}_{d}}$$

The 6 different errors $E_{P}^{+},E_{P}^{-},E_{A}^{+},E_{A}^{-},E_{D}^{+},E_{D}^{-}$ , for high and low activation, separately for the three PAD dimensions, computed on our calibration data are the following:

$$Pleasure:\;E_{P}^{+}=0.138\;;E_{P}^{-}=0.098$$

$$Arousal:\;E_{A}^{+}=0.071\;;E_{A}^{-}=0.134$$

$$Dominance:\;E_{D}^{+}=0.052\;;E_{D}^{-}=0.020$$

Then, at run time, after the calibration, user-customized thresholds will be computed as deviation from his/her median.

$$T_{d}^{+}=M_{d}+E_{d}^{+};T_{d}^{-}=M_{d}-E_{d}^{-}$$ (1)

### 5.6 Overall control of avatar sensitivity

As mentioned earlier, a key objective of the system is to control the cobot and avatar’s intervention frequency. Following the calculation of six personalized thresholds (one for each PAD dimension–pleasure, arousal, dominance) based on user calibration, an additional control mechanism is implemented. This involves defining a global multiplier K and using it to compute modulated errors:

$$E_{\prime +}^{d}=E_{d}^{+}*K;E_{\prime -}^{d}=E_{d}^{-}*K$$

Reapplying Equation 1, we define 6 re-modulated thresholds $T^{{\prime +}_{P}},T^{{\prime -}_{P}},T^{{\prime +}_{A}},T^{{\prime -}_{A}},T^{{\prime +}_{D}},T^{{\prime -}_{D}}$ . From another perspective, the inverse of K can be seen as a more intuitive measure of *sensitivity* S=1/K .

At run-time, when a triplet of samples $p_{d}$ is received, an intervention is triggered if *all* the three PAD signals go either above $p_{d}>T^{{\prime +}_{d}}$ or below $p_{d}<T^{{\prime -}_{d}}$ . The combination of low/high activations defines an *activation code*. For example, +P+A-D denotes a combination of high pleasure, high arousal, and low dominance.

### 5.7 Finding the ideal global sensitivity

After determining a set of thresholds for positive and negative PAD triplets from a single S multiplier, the problem to address now turns to be: “What would be the optimal sensitivity S for the cobot-avatar system?”.

To determine an appropriate intervention frequency for the covatar (around 5 interventions per 30-minute session), we analyzed our system logs. We specifically examined how often interventions would be triggered at various sensitivity levels based on individual data samples. These intervention counts were then averaged across all participants. The results (shown in Table 3) reveal that lowering the sensitivity level reduces the average number of interventions for each PAD dimension (pleasure, arousal, dominance). For example, the average activation count for individual dimensions drops from over 450 to less than 60 as sensitivity decreases. However, we also investigated co-occurrences - how often all three PAD dimensions would exceed their thresholds simultaneously. With a multiplier value (K) of 2.0, the maximum average for these simultaneous interventions was 10. This number significantly decreased to just 0.21 with a K value of 2.4, and then dropped to zero entirely. These findings led us to reject our initial assumption that PAD channel activations necessarily happen at the same time. In other words, the system cannot rely solely on simultaneous threshold breaches across all three dimensions to trigger interventions.

**TABLE 3:** Top: average number of activations for P, A, D, and all signals. Bottom: activations in a time window, normalized by its size $\left(s^{-1}\right)$ .

K	P	A	D	ALL
2.0	557.14	657.29	479.29	10.79
2.1	484.71	577.93	429.14	4.07
2.2	418.21	518.00	389.50	2.29
2.3	362.57	473.50	351.43	1.21
2.4	313.29	427.07	314.93	0.21
2.5	267.79	381.07	280.29	0.00
2.6	223.57	323.57	250.86	0.00
2.7	187.86	259.86	223.50	0.00
2.8	159.93	187.93	201.29	0.00
2.9	127.71	118.29	179.36	0.00
3.0	102.93	70.71	161.71	0.00
3.1	78.14	43.14	144.50	0.00
3.2	56.14	33.14	127.64	0.00
3.3	43.64	30.79	111.07	0.00
3.4	32.00	27.86	99.07	0.00
3.5	22.29	26.29	87.07	0.00
3.6	14.07	23.71	77.21	0.00
3.7	7.86	21.71	66.50	0.00
3.8	6.00	20.21	60.07	0.00
3.9	4.36	19.00	53.79	0.00

	Time window ( W , secs)				
**K**	**5**	**30**	**60**	**120**	**300**
2.0	20.56	25.25	21.87	17.57	10.28
2.1	14.84	20.66	18.73	14.98	9.17
2.2	9.77	15.88	16.07	13.72	8.46
2.3	6.83	12.34	12.88	12.23	8.09
2.4	4.71	9.61	9.58	8.92	6.59
2.5	4.06	9.11	9.11	8.37	6.34
2.6	3.44	7.83	7.29	6.67	5.51
2.7	3.06	6.75	6.73	6.21	4.94
2.8	2.57	5.30	5.78	5.35	4.63
2.9	2.11	3.93	4.95	4.88	4.23
3.0	1.50	3.00	3.89	3.74	3.10
3.1	0.97	2.23	3.43	3.42	2.84
3.2	0.94	1.89	2.89	2.77	2.34
3.3	0.44	1.35	1.51	1.66	1.78
3.4	0.03	0.73	0.99	1.49	1.45
3.5	0.03	0.66	0.95	1.35	1.32
3.6	0.00	0.12	0.36	0.62	0.63
3.7	0.00	0.00	0.00	0.00	0.00

This concept is further supported by existing research in the field. Studies have shown that emotions manifest differently on people’s faces, exhibiting variations in activation delays, persistence, and relaxation times. This asynchronous nature is reflected in the PAD signals, which can become misaligned.

To account for this, the system identifies an activation code at time t by looking back in time within a defined window of size W . Here, each PAD dimension (pleasure, arousal, dominance) is evaluated separately. The scan progresses backward through the time window, searching for a threshold exceedance in any given dimension. If a threshold is crossed within the window, or the entire window is exhausted without finding an activation, the search concludes. The *PAD activation code* is only set if activations are detected within the window for all three channels. In essence, the system acknowledges that PAD activations may not always occur simultaneously, and it adjusts its intervention logic accordingly.

Our final data analysis focused on identifying the ideal time window size (W) for activation detection. To address this, we once again employed simulations. We calculated the average number of PAD activations across all users for various window sizes. The results are presented in Table 3 (bottom). As anticipated, a larger window size leads to an increase in the average number of activations. This is because “older” activations remain within the window for a longer duration as the reference point for comparison (current time) advances. The table also reveals that the number of activations per second increases steadily until a window size of 120 s is reached, and then starts to decline. This can be explained by the fact that with an excessively large window, newly activating samples begin to obscure older ones. In other words, looking too far back in time does not provide additional valuable information. Interestingly, consultations with psychologists also suggest that a time window between 30 and 60 s is sufficient to capture facial expressions linked to emotions that have not yet subsided.

**FIGURE 6:**
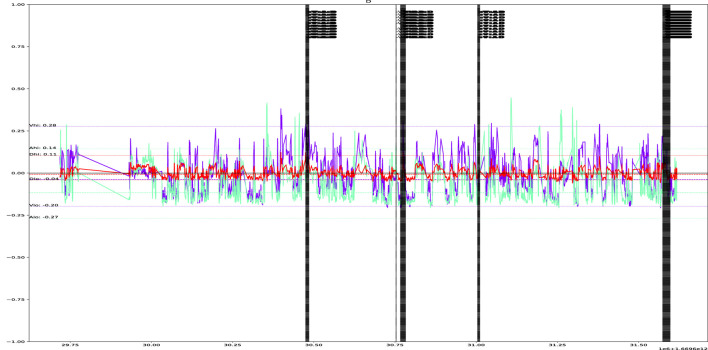
Example of PAD streams over 30 min and their activations (vertical lines), for a single user, with W=30 seconds and K=2 .

Figure 6 depicts an example activation sequence for a single user. The figure highlights that activations tend to occur in “clusters.” This is because when examining the data on a sample-by-sample basis, activations remain active until they exit the defined time window. Interestingly, the figure also reveals five distinct clusters, a pattern commonly observed across most users. This suggests that the combination of a time window size (W) of 30 s and a multiplier value (K) of 2.0 is a well-suited configuration for our experiments.

### 5.8 Synchronization with the work task

Our final design decision focused on determining the most appropriate timing for cobot and avatar interventions. Triggering interventions immediately upon detecting an activation code could lead to disruptive, consecutive interruptions, especially during periods of high user concentration.

To address this concern, we opted to synchronize interventions with the assembly cycle. The system discards any historical data received from the emotion sensor (SSI) at the start of each cycle. It then accumulates samples throughout the assembly task (lasting approximately 50 s). Only after the user successfully completes their assembly by joining it with the cobot’s (a moment of potentially high focus), does the system evaluate the data for PAD activation codes and potentially trigger an intervention. In essence, the system only reacts to excessive PAD values measured during the assembly cycle that was just concluded.

For our specific use case, the optimal time window range of 30–60 s aligns well with the duration of the assembly task. However, different application domains might necessitate alternative intervention timing strategies.

## 6 Study 1: validation of the theoretical assumptions of the BASSF model

### 6.1 Research questions and hypotheses, study 1

We conducted a controlled study to systematically assess the theoretical transparent assumptions underlying the BASSF Model. We empirically tested the possibility of representing flow in the PAD space, to be able to provide continuous input to the BASSF model and enable socially interactive behavior for the covatar. To achieve this, we needed to define the relationship between flow and PAD. Hypotheses H1 to H3 test this relationship:

We conducted a controlled exeprimental study to assess the theoretical underpinnings of the BASSF model. This investigation focused on empirically testing the feasibility of representing Flow within the PAD framework. We aim to enable continuous input on the Flow levels of the user for the BASSF model and ultimately facilitate the generation of socially interactive behaviors for the cobot agent. This objective involves defining the relationship between Flow and the PAD dimensions. Hypotheses H1 to H3 were formulated to address this specific relationship: **H1:** Self-reported Self-Efficacy will predict flow. **H2:** Dominance will predict Self-Efficacy. **H3:** There will be a significant linear relationship between pleasure, arousal and dominance as predictors and flow as outcome.

In **H1**, we examine the theoretical relation between Self-Efficacy (Matthews et al., 2022) and flow. We consider the association between Self-Efficacy’s and the subjective perception of challenge-to-skill ratio. In **H2**, we test the relationship between Self-Efficacy and dominance, which would validate representing self-efficacy through the Dominance dimension in PAD (Gilroy et al., 2009). Finally, following the prediction formulated by Gilroy et al. (2009), we **H3** test the relation of all three PAD dimensions to flow and its representation in the PAD space. We test the specific assumption that flow can be represented in the Octant Space as quadrant +P,+A,+D.

To gain deeper understanding of emotional experiences associated with Flow in a production line setting, we employed a semi-structured interview format. The interview focused on an exploratory investigation guided by the following research question: “What emotions do participants experience during the task, and what strategies do they employ for emotional regulation?”

This study utilized a mixed-methods design to investigate emotional experiences during assembly tasks. Participants collaborated with an interactive cobot agent on the task, which was manipulated to have two phases: slow (inducing boredom) and fast (inducing stress). Following each work phase, participants reviewed video clips from their experience and completed a battery of six self-report questionnaires. These questionnaires assessed their affective state (emotions), Flow experience, and perceived Self-Efficacy during the task Section 6.3). A semi-structured interview on their emotion regulation strategies was performed by Nicora et al. (2023) in between the questionnaires.

### 6.2 Participants and procedure, study 1

To ensure optimal task durations and system functionality, a pilot test was conducted with two participants. Subsequently, data collection proceeded with the final sample of 20 healthy adult volunteers (12 male, 8 female; age range: 25–48 years). It is important to note that the semi-structured interview component of the study was only implemented with the initial four participants.

Following an introduction and task familiarization, individual participated in the experimental sessions (50 min). The sessions were structured as follows: Phase 1 - slow phase (30 min), followed by Phase 2 - fast phase (20 min). To make the transition between Phases 1 and 2 seem natural, and evoke more pronounced emotional responses, the transition between phases was implemented through a staged system “failure.” 30 min after the beginning, and the Cobot ceased working. The operator would then intervene, pretending to be “fixing” the failure. The operator modified the setup and instructed the participant to adapt to the robot’s increased pace.

At the end of Phase 2, participants were escorted to a separate room. The experimenter and each participant reviewed six video clips of the participant working with the Cobot. All participants completed a questionnaire for each scene, while the semi-structured interview was additionally conducted with the first four participants. The total assessment time was approximately 20 min for questionnaires only and 45 min when the interview was additionally conducted.

To isolate and evaluate specific assumptions of the BASSF model, a simplified version of the model was employed in Study 1. Covatar interventions were pre-programmed to address expected emotional experiences at paricular times in a sequential order: Overwhelming Boredom (O-Boredom), Anxiety, and Underwhelming Boredom (U-Boredom). During the slow task phase, the first intervention targeting Overwhelming Boredom (O-Boredom) commenced 15 min after the experiment began. The initial intervention for Anxiety was introduced 5 min into the slow task phase, followed by an Overwhelming Boredom (O-Boredom) intervention 7 min later (12 min after the start).

### 6.3 Instruments and interview, study 1

To assess pleasure, arousal, and dominance, we used the semantic differential (Mehrabian and Russell, 1974). This comprises six adjective pairs with nine spaces in between for each dimension. To indicate emotional experience, participants mark position of on the line. Flow was measured using the flow short scale (Engeser, 2012; Rheinberg et al., 2003), which includes ten subcomponents. State Self-Efficacy was measured with a single item (Matthews et al., 2022).

The semi-structured interview required participants to watch the six recorded videos of the three pre-selected interventions. Participants were guided to describe their emotions and thoughts with regard to the task, the avatar, and effects of the intervention. To analyze the transcripted reports, we performed a deductive-category analysis (Mayring, 2014). Two category systems were used to code the interviews, encompassing affective experience and emotion regulation. To see whether the quantitative and qualitative descriptions would align, the categories of affective experience were related to the Octant Space. We added two categories to the Octant Space to represent Shame and “not inferable” (NI), as we expected shame and its regulation to arise.

The emotion regulation coding system included: cognitive reappraisal, distraction, acceptance, rumination, and disengagement (Elison et al., 2006; McRae and Gross, 2020), which are the most common regulation strategies, along with three out of four additional strategies from the compass of shame: attack other, attack self, and avoidance. The fourth strategy, withdrawal was not possible in the experimental without droping out of the experiment (Nathanson, 1994; Elison et al., 2006). We further defined avoidance, disengagement, and distraction as follows. Distraction defines an act where the participant is aware of a negative stimuli, and refocuses attention to a more pleasant stimulus (McRae and Gross, 2020). Avoidance and behavioral disengagement adjust the relevance of the stimuli for the individual, but behavioral disengagement involves willful and conscious withdrawal of efforts related to the task difficulty, whereas in avoidance there is no acknowledging of the negative experience, and still the relevance of the stimuli is reduced, for instance, by loosing interest (Elison et al., 2006; Nathanson, 1994).

### 6.4 Experimental setup BASSF evaluation, study 1

The experimental setup described in Section 3.1 was used. The assembly task was modified for the purposes of the testing, to isolate important aspects of the BASSF model. Two experimental phases were prepared, in order to elicit different reactions by the participants. The first boring phase made use of all assembly capabilities of the cobot. The Cobot looked for the parts by use of a detection camera, picked them up, and assembled them in order to bring them to the operator for the final combined assembly. 50–60 s are needed for this, and this time is more than enough for the operator to complete their part of the assembly, which may lead to boredom and frustration. A second fast phase was rather explicitly aimed at eliciting stress. In the right handside of Figure 2 one can see how the spare components on the cobot’s table are substituted by an array of preassembled parts, so that the cobot can reach a predefined position to get the preassembld part to bring it to the user. This lasts 10–15 s, and leaves no time for the operators to assemble their part. Seeing how the Cobot has to wait on them is a result of this. Still participants had to assemple as many complete parts as possible in the available experiment time. They were required to have finished one whole piece before working on a new one.

### 6.5 Quantitative results, study 1

Descriptive Statistics for all measured variables can be found in Table 4. For hypotheses 1 and 2, the connection between Dominance, Self-Efficacy, and Flow was analyzed using Mixed Effect Models with Random Intercept, due to the nested structure of the data. The participant was used as a grouping variable to account for the correlations between repeated measurements (Detry and Ma, 2016). The tests were done with and without centering around the group mean (Enders and Tofighi, 2007). Centering did not affect the results on the relationship between Dominance and Self-Efficacy. However, in the relationship between, Self-Efficacy and Flow, centering had a significant impact on the results; Self-Efficacy was a significant predictor of Flow, while centered Self-Efficacy was no significant predictor of Flow (see Table 5).

**TABLE 4:** Summary statistics of all constructs measured with the questionnaire. Round brackets in the “Min” and “Max” Columns denote theoretically possible min and max values.

Summary statistics, study 1					
Construct	Mean	Median	SD	Min	Max
Pleasure	0.078	0.063	0.304	−0.833 (−1)	0.833 (1)
Arousal	−0.099	−0.125	0.292	−0.75 (−1)	0.583 (1)
Dominance	0.134	0.167	0.31	−0.792 (−1)	0.833 (1)
Flow	4.995	5	0.687	2.9 (1)	6.5 (7)
Self-Efficacy	4.042	4	0.614	2 (1)	5 (5)

**TABLE 5:** Note: Se = self-efficacy, D = Dominance, ID = participant; sig: p<0.001 ‘***’; p<0.01 ‘**’; p<0.05 ‘*’; p<0.1 ‘.’

Results of hypothesis 1 & 2, study 1				
Formula		SE ∼ D (Cent.) + (1 — ID)	Flow ∼ SE (Cent.) + (1— ID)	Flow ∼ SE + (1— ID)
REML criterion		135	175.7	171.5
N observations		120	120	120
N groups		20	20	20
Fixed effects				
Intercept	Estimate	4.042	4.995	3.976
	SE	0.118	0.133	0.421
	t-stat	t (19) = 34.14	t (19) = 37.678	t (106) = 9.437
	p	*p* < 0.001 ***	*p* < 0.001 ***	*p* < 0.001 ***
Coeff	Estimate	0.686	0.099	0.252
	SE	0.181	0.112	0.101
	t-stat	t (99) = 3.8	t (99) = 0.881	t (115) = 2.512
	p	*p* < 0.001 ***	*p* = 0.381	*p* = 0.013 *
Method of *t*-test		Satterthwaite’s method	Satterthwaite’s method	Satterthwaite’s method
AIC		143.039	183.719	179.511
BIC		154.189	194.869	190.661

**FIGURE 7:**
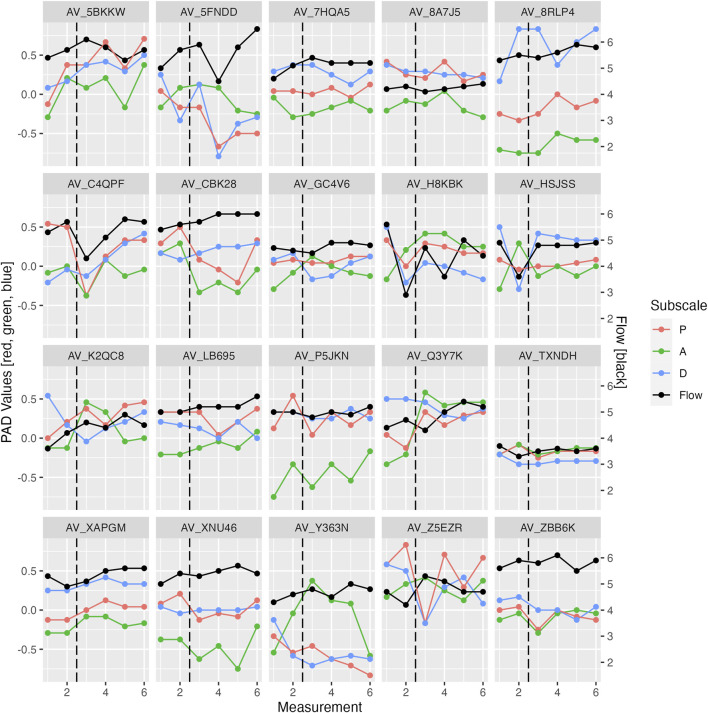
Flow and PAD values over time for each participant, Study 1. The dotted vertical line represents the change from the slow to the fast phase.

For hypothesis 3, several mixed effect models were used to examine the relationship between PAD and Flow (for a visualization see Figure 7). Multiple regression was not useable due to the nested structure of the data. Centering did not affect the significance of the results. The mixed effect models showed no significance for any one of the predictors when used together (see Table 6). However, strong correlations between the predictors were found (Pleasure & Arousal: r = −0.59; Pleasure & Dominance: r = −0.55, Arousal & Dominance: r = 0.54). When the predictors were on their own, Dominance was a significant predictor (see Table 6).

**TABLE 6:** Note: P = pleasure, A = Arousal, D = Dominance, ID = participant; sig: p<0.001 ‘***’; p<0.01 ‘**’; p<0.05 ‘*’; p<0.1 ‘.’

Results of Hypothesis 3, Study 1					
Formula		Flow ∼ P + A + D + (1 — ID)	Flow ∼ P + (1 — ID)	Flow ∼ A + (1 — ID)	Flow ∼ D + (1 — ID)
REML criterion		171.1	171.6	175.4	170.5
N observations:		120	120	120	120
N groups		20	20	20	20
Fixed effects:					
Intercept	Est.	4.995	4.995	4.995	4.995
	SE	0.133	0.133	0.133	0.133
	t-stat.	t(19) = 37.678	t(19) = 37.678	t(19) = 37.678	t(19) = 37.678
	p	*p* < 0.001 ***	*p* < 0.001 ***	*p* < 0.001 ***	*p* < 0.001 ***
Coeff.	Est.	P: 0.274	0.400	-0.002	0.461
		A: 0.003			
		D: 0.367			
	SE	P: 0.274	0.209	0.199	0.211
		A: 0.254			
		D: 0.269			
	t-stat.	P: t(97) = 1.001	t(99) = 1.913	t(99) = -0.011	t(99) = 2.182
		A: t(97) = 0.016			
		D: t(97) = 1.364			
	p	P: p = 0.319	*p* = 0.059 .	*p* = 0.992	*p* = 0.032 *
		A: p = 0.988			
		D: p = 0.176			
Random effects:					
Intercept	Est.	0.326	0.325	0.324	0.326
	SD	0.571	0.570	0.570	0.571
Res. Var.	Est.	0.156	0.157	0.163	0.155
	SD	0.395	0.396	0.403	0.394
Method of t-test		Satterthwaite’s method	Satterthwaite’s method	Satterthwaite’s method	Satterthwaite’s method
AIC		183.055	179.622	183.352	178.535
BIC		199.780	190.772	194.503	189.685
VIF		P: 1.73			
		A: 1.70			
		D: 1.61			

An in-depth investigation of the relationship of PAD and Flow for each time frame was attempted, but the statistical power for n = 20 data points was too low (below 0.8).

### 6.6 Qualitative results, study 1

After performing deductive category assignment on the dataset, we calculated several descriptive statistics to better understand the results of the two main category systems: “Affective Experience” and “Emotion Regulation” (see Table 7).

**TABLE 7:** Descriptive Results for Deductive Category Assignment, Study 1. Note that the proportion of observation is an estimation. Timestamps, Ids, comments, translated passages, and multiple codings have not been removed.

	Affective Experience	Emotion Regulation
Mean Obs. per Category	3.7	1
SD	4.27	1.12
Min. Obs. per Category	0 (“Flow”, “Awe”)	0 (“Attack Self”, “Acceptance”, “Rumination”, “NI”)
Max. Obs. per Category	14 (“Relaxation”)	3 (“Cognitive Reappraisal”)
Proportion of Obs.	33.72%	9.45%

The high SD in the Affective Experience column indicates that the distribution of observations across categories within each system is centered around some variables, which is unsurprising since all participants participated in the same experimental setup.

The affective experience and regulation strategies will be summarized here: participant A (also called AV_C4QPF as reference for Figure 7) felt relaxed at first during the slow phase. When the speed increased at the start of the fast phase, the participant started feeling many different feelings such as anxiety, o-boredom, hostility, shame, and hopefulness. The participant felt stressed because they^1^ are slowing the assembly down. They decided to reduce their efforts and to go at their own rhythm. “It is enough for it to be repetitive; I do not need it also to be to be fast.” This behavioral disengagement did not, however, instantly alleviate the pressure. Before it caused relaxation, it caused feelings of shame for not being able and not willing to keep up with the robot.

Participant B (AV_GC4V6) felt relaxation and u-boredom. They unintentionally distracted themselves by thinking about their work, which led to them doing a mistake while assembling. When the fast phase started, the participant started to feel stressed by the speed of the robot. The participant then tried to reappraise the situation: “I was like, no, this is my job and the robot is just a robot. So he has no feeling he can wait. […] Also because I was kind of telling myself that I had to do the most difficult part like matching the gears going, with the clips and stuff.”^2^ The participant kept their speed and reported feeling more relaxed after half of the “fast phase” had passed.

Participant C (AV_LB695) described only feeling relaxed and no boredom in the beginning because they felt they were doing a purposeful commitment that would eventually end. In the “fast phase”, the participant described not feeling rushed: “I thought that since the number of movements that the machine had to do was smaller then what I had to do was just completing the task earlier than I. […] It was just waiting for me, but it was not a problem.” Notably, when asked how they dealt with unsolvable tasks, the participant believed that in such a case, the blame lies with the person who gave them the task, not them. (“Yeah, probably, especially if it was in a real situation then I would have thought that the whole planning of the operation was bad because even if I strived, I would not have managed to be so quick and be always on time for the robot. So yeah, I would be angry in case it would always be like that.”)

Finally, Participant D (AV_ZBB6K) described themselves as being relaxed. However, they distracted themselves by thinking about how to improve the work cell and the task. This tactic ended around the middle of the “slow phase” when Participant D started to feel bored. This was coupled with some short interruptions where the system experienced errors that needed to be fixed. When the “fast phase” started, the participant did not increase their speed. When asked about the reason for this, the participant said they did not care anymore. Further investigation no reason for this could be found as the participant said they did not know why they felt that way. Therefore, we categorized this as a case of avoidance.

While the self-ascribed feelings of the participants usually matched the results of the semantic differential. 13 times one of the self-ascribed states matched the result of the semantic differential. However, six times there was a mismatch. Most often (4 of 6) it involved the ascription of awe and hopefulness from the semantic differential. In the interviews, they were described as relaxation (2 of 4) but also as anxiety.

### 6.7 Discussion on the BASSF evaluation, study 1

Based on the mixed results on H1, a clear conclusion could not be reached; further research is necessary (see below for an in-depth discussion). H2 showed significant results and is therefore accepted. H3 showed no evidence of a connection between PAD as a whole and Flow. The hypothesis will be rejected. However, the connection between Dominance and Flow is significant and will be investigated further.

Uncentered Self-Efficacy predicted Flow, which strengthens the association between Self-Efficacy and the challenge-to-skill ratio, which was known to be a predictor of Flow (Moneta, 2021). However, group-centered Self-Efficacy is not. One possible explanation for this would be that Self-Efficacy remained relatively stable, and the centering thereby caused many zero values. While the scale was validated (Matthews et al., 2022) it contained only one item, and therefore only integer values were possible. A possible solution to this would be to use a scale that contains more than one item or increase the sample size.

Dominance predicted Self-Efficacy. This stresses the role of Dominance in the PAD model, especially for task-related settings. Continuous monitoring of Dominance could capture how workers feel about a task and whether they see themselves as capable of handling the task or if they feel overwhelmed by it and plan interventions for wellbeing. Additionally, Dominance has the strongest connection to Flow from all models tested. The model that only used Dominance had the lowest AIC and BIC and therefore seem to offer the best fit to the data. From all known variables in this experiment, Dominance is best suited for Flow estimation and should be included in its modeling.

The results on the connection between Flow and PAD are mixed. Surprisingly, and against previous theoretical definitions (Engeser et al., 2021; Abuhamdeh, 2021), the results show that pleasure and arousal were not connected to Flow in this experiment. Our results suggest that the presence of pleasure in the model strongly influences the estimations for Dominance, which is not unlikely due to the strong correlation between pleasure, arousal, and Dominance.

The lack of significance of pleasure on Flow contributes to the discussion of enjoyment in Flow (Abuhamdeh, 2021). Given the assumption that Flow is an enjoyable experience, an increase in Flow should have also led to an increase in pleasure. We found no evidence for this claim. However, we can also not rule it out based on our results, as it only describes a partial one-directional relationship, which our model cannot test. Increases in Flow predict increases in enjoyment; however, increases in enjoyment do not predict the Flow level.

In the literature, high arousal was associated with Flow (Engeser et al., 2021). In our scenario, this would association would be hard to support, as high levels of arousal can be associated with stress, which is more likely to occur in shifts of up to 8 h.

### 6.8 Discussion of qualitative results, study 1

All participants described the tasks as relaxing and positive while acknowledging their repetitiveness. This positive feeling faded in the fast phase when the task became more stressful for 3 of the 4 participants.

After the introduction of the fast phase, none of the participants were able to keep up with the robot. A fluctuation of different emotions was noticed by 3 of the 4 four participants, which may indicate that they were not sure how to properly adapt to the situation and is an indicator of stress. However, all participants found a way to deal with the stress and reached a constant state towards the end of the experiment. This was relaxation for three of them and hostility and boredom for one of them. All participants that felt stressed by the speed of the covatar reduced its intensity by regulating their emotions through reappraisal, avoidance, or disengagement. However, two reduced their performance, and one did not enjoy their affective state towards the end. This indicates that while participants were able to deal with stressors, their strategies used did not always yield the most optimal results in terms of productivity and wellbeing in the production line.

Boredom was regulated less often than stress and anxiety. However, this might have been because participants knew about the length of the experiment and thereby did not feel any pressure to regulate boredom. Still, all regulation strategies used were not optimal as they distracted themselves from the task, leading to errors, and were not sustainable for longer periods. This highlights the importance of positive guidance in emotion co-regulation.

## 7 Study 2: first lab evaluation of the overall system

### 7.1 Experimental setup, study 2

In a first lab evaluation of the overall system, Study 2, 12 volunteers where recruited among the employees of the CNR institution in Italy, or among students of the near-by University, both in Lecco, Italy. They aged from 19 to 33 and were approximately balanced by gender. The volunteers worked with the covatar for 1 week, approximately 3.5 h a day. Seven vollunteers had previously worked with the basic cobot (without the Avatar and the BASSF model), and five were new volunteers and had no previous experience with cobots. A few days later (no longer than a week), they completed the Mental Health Continuum (MHC-SF) (Keyes, 2005), to assess their emotional, social and psychological wellbeing. In addition, participants completed a newly designed questionnaire to assessed their work, experiences and the covatar on a Likert-type scale from never (1) to always (5). They were also asked, in open questions, to list positive and negative sides of the MindBot platform and suggestions for possible enhancements. At the end, the participants assessed their attituded toward robots, filling out the Negative Attitude toward Robots Scale (NARS) (Nomura et al., 2006).

### 7.2 Log analysis, user-experience and discussion, study 2

**FIGURE 8:**
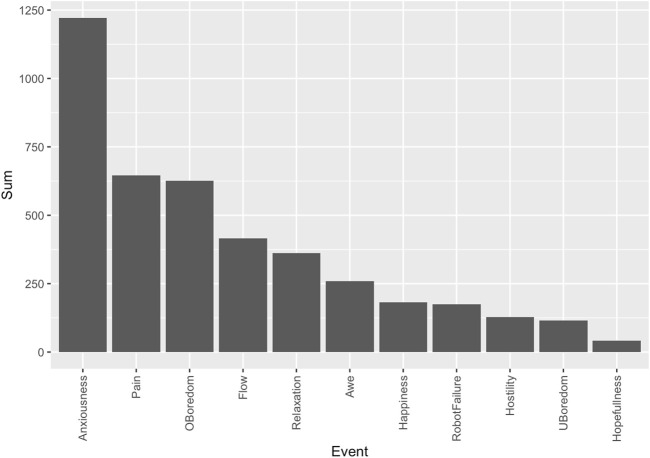
Sum of the number of events over all users, grouped by event type.

A log analysis of the 12 participants showed some first promising results. Figure 8 shows that (besides pain, which is external to the Flow space) productive challenge (productive Anxiousness and Flow), as well as over-challenge boredom were detected in the PAD space. Flow experiences are rare (Bartholomeyczik et al., 2023) and heavily dependent not only on individual differences, but also on situational characteristics (Fullagar and Kelloway, 2009), this is a considerable achievement. High productive Anxiousness aligns with the theoretical basis of the BASSF model, that consider the perceived skill-to-challenge ratio (McRae and Gross, 2020) to reduce perceived high challenge through self-compassion and transform it into manageable and welcome challenge. With regard to over-challenge boredom, this results constitutes extra support for symptoms of boredom relating rather to over-challenge, as discussed above. Reacting with interventions to motivate the workers to perform better would be counterproductive for their wellbeing.

### 7.3 Assessment of workers’ experience, study 2

The assessment of the workers’ experience also supports these preliminary results. Participants reported overall good levels of mental health, especially psychological and emotional wellbeing. Specifically, five participants had complete mental health - flourishing in life (Keyes, 2002), - five were moderately mentally healthy and two of them had low mental health - languishing (Keyes, 2002). Working with the covatar was evaluated as a positive experiences during real time job tasks, including Flow, but also task efficiency.

Quantitative analyses showed that workers did not find it difficult to complete the task. They experienced high levels of competence and autonomy, felt mostly safe, relaxed, and reported average levels of satisfaction, being concerned or irritated and realised that they did not have control over the covatar. The workers also noticed that the covatar collaborated with them, enabled them to work fluently and improved their effectiveness. Feeling safe and competent may have contributed to psychological wellbeing. Participants with more negative emotions toward robots felt more tensed. They subjectively perceived fewer covatar reactions to their behavior and less support during deadlocks, and concluded that covatar did not increase their efficiency.

In addition most of the workers noticed and liked that the covatar was trying to adjust the work speed with operator’s fatigue and that it warned when it could not find the parts. Common negative experiences with the covatar rather relate to operating errors of the system that led to feeling tensed and perceiving reduced effectiveness, which may undermine positive experiences of high competence and autonomy. Some participants reported that the covatar occasionally made incorrect conclusion about their physical state (fatigue). They also complained about the figure of the Avatar, as well as about the lack of diversity in Avatar messages and interventions.

Finally, an analysis using the Experience Sampling Method (ESM) (Csikszentmihalyi and Larson, 1987) showed slightly increased self-reported emotional experiences related to higher challenge including Flow, and less low-challenge experiences, including relaxation, for workers using the covatar. Most importantly, when considering fluctuations over the total period of interaction with the covatar for each Flow dimension, these effects are higher than average, and workers also reported more satisfaction, but less under-challenge boredom and constraint. An analysis of real-time physiological data (heart rate and movement data: steps, activity, accelerometer data) collected by a commercial wearable and connected to Mindstretch platform developed by BioRICS N.V., estimated the mental energy used during Flow and Control experiences with the covatar and associated it with more mental energy use.

### 7.4 Discussion of the overall BASSF evaluation, study 2

Overall, the higher Flow and productive Anxiousness automatically detected during the interaction with the covatar by the BASSF model can be positively contrasted to the self-reported under-challenge boredom when not using the cobot, and aligns both with the results of the ESM and the energy level use. This contrasts point to a healthy reduction of under-challenge while not increasing stress. Moreover, Flow and Control were the two ESM variables that showed higher energy use levels. This is an interesting result because Control is associated with Self-Efficacy and, hence, the Dominance dimension of the PAD space. As such, this supports the results from the BASSF model evaluation, namely, that the Dominance is the best predictor of Flow independent of the other two dimensions (pleasure and arousal).

The results in general provide a validation for the modelling of the Flow space in the PAD space for continuous monitoring, however, Flow experience should be measured through Dominance alone, whereas Pleasure and Arousal are useful for the prediction of the other states in the PAD space that point to the right intervention in order to support Flow. At the same time, the results support these interventions that were theoretically conceived for the BASSF model and concentrate on fostering the perception of a balanced skill-to-challenge ratio through awareness interventions that support the workers’ focus on, or away from aspects related to the skill-to-challenge perception. In combination with lower energy levels use in anxiety states and without the covatar, the result point to higher wellbeing when using the covatar which fosters a balanced skill-to-challenge ratio, despite subjective self-reports among workers with negative perceptions of robots. Since workers also reported not controlling the Avatar although they found the task boring, it seems like the feeling of productive challenge can be rather put down to the socially interactive covatar.

With regard to the Avatar figure, the perception of it as agreeable or not, much as it aligns with diversity considerations, it is possibly related to cultural preferences. Nevertheless, there were no differences in worker experiences with regard to gender, that might be due to the gender-neutral avatar. Also, diversity of messages is an easy thing to accomplish, as the system developed an interactive agent based on theoretical constructs. Hence, the interventions defined in the BASSF are not scripted and can be verbalised in a variety of ways, while taking systemic and cultural needs of companies into account. VSM (Gebhard et al., 2012a) can be used also by non-programmers to this end. Finally, cobot errors should undoubtedly be avoided, but the addition of the avatar improves the overall emotional experience despite the high number of cobot failures.

## 8 General discussion

The presented method shows the advantages of: i) combining avatars and cobots through using the BASSF model, and ii) the post-processing technique for social signals interpretation that feed the collaborative interaction with the covatar system.

### 8.1 Technical implications

The method tackles several technical challenges:

• Inconsistent Signal Quality: it addresses issues like missing data points and fluctuations within the PAD signals.
• Personalized Baselines: it accounts for individual user differences by requiring a calibration process for each person.
• Threshold Definition: it establishes a framework for defining activation thresholds that trigger specific behavioral responses from cobot and avatar.
• Temporal Misalignment: it addresses the potential for misalignment between the PAD data streams.

A key advantage of this method is its domain-specific customization. By collecting data from a relatively small group of users (14 participants in Study 1 were used for system tuning in this case), the system can be adapted to a particular domain. This data collection requirement is significantly less expensive compared to the vast amount of labeled data needed for training neural network-based, end-to-end approaches. The described post-processing method played a crucial role in the overall system evaluation.

We believe the post-processing strategy developed for PAD predictions presented in this work can be valuable for future research. This method and the results we’ve shared can help researchers more efficiently address similar challenges that are likely to arise when analyzing user emotions using hardware sensors.

Furthermore, we hope to encourage future research in social signal interpretation to move beyond simply extracting “raw” data. By incorporating post-processing strategies like the one described here, the social signal processing community can make significant advancements in developing real-world applications. In general, we provided a round and detailed account on how to create a more sociable industrial environments using socially interactive cobots, and how this can help to support workers’ wellbeing and Flow, that can at the same time maintain or even increase worker involvement in a safe and enjoyable manner.

### 8.2 Studies implications

Somewhat surprisingly, the findings of Study 1 suggest that the Dominance dimension of the PAD model plays a crucial role in predicting Flow. Furthermore, the results of Study 1 highlight the importance of guidance in emotion co-regulation, as some strategies used by participants negatively impacted their wellbeing and productivity. This is the first attempt to reevaluate the significance of the Dominance dimension of PAD.

The results of the evaluation of the overall system, Study 2, support the BASSF model, provide insights into the effects enhancing industrial cobots with socially interactive avatars, and support their use for wellbeing. Concretely, Flow experiences are highly dependent on individual workers, their day-to-day experience and situational characteristics. Since situational characteristics are very hard to adjust in industrial settings, it is very important to apply adaptive and personalised socially interactive systems like the one developed here. Such systems can monitor the workers’ wellbeing and escort them through their emotional fluctuations like a co-worker would do. They may provide a social context that is missing in the highly automatised industrial world.

Personalised avatars depicting higher external similarity to workers were beyond the scope of this project, but may be an alternative to using gender-neural Avatars that were negatively assessed by users. Avatar similarity is being investigated with regard to increased affinity and self-compassion with positive results (Alves da Silva et al., 2023). Moreover, it is very important to be able to test the differential effects of the different interventions of the covatar and even tear them apart from the verbalisations. We reported here how the BASSF model allowed us to test and understand the results of the socially interactive covatar and also relate them to other results and to theory. Still, the results point to the need to include explicit Covatar feedback to workers about their productivity and comparing that to the current implicit co-regulation.

In general, the results from Study 1 align with and are validated by the results of the evaluation of the overall systems in Study 2, and show an improvement of the user emotional experience as a whole.

Finally, both studies have limitations. The small sample size and the predictor correlation limited the statistical exploration of the Flow-PAD relationship in detail. The interviews provide qualitative insights into participants’ experience, that helps to understand the quantitative results. Similarly, the results in Study 2 give a detailed view into the user experience with regard to the specific task. It uses an exemplary approach assessing user wellbeing through the combination of objective and subjective measures, situative evaluations, and overall perceptions. Furthermore, the avatar was mainly responsible for delivering the BASSF model through the defined interactions, and as such the current positive results show a clear promise for the added value of the covatar. However, the results of the subjective experiences of the participants, in particular, do not allow drawing conclusions on whether the experience is improved when using the system with versus without the avatar, since only seven from 12 participants had used the system without the avatar. However, the results of subjective experiences of the users in both studies, although precious for design considerations, should be interpreted with caution as cannot be generalized due to the small samples. Also cobot failures that also influence the user experience should be reduced in order to evaluate the Avatar alone.

On the whole, further validation is needed to consolidate the results and theory-based interpretations. This can look into concrete interventions, and provide insight into differential co-regulation reactions, e.g., based on the dichotomous cause of boredom (overwhelming versus underwhelming experiences). Also, future work may test replicability and scrutinize the relations of the main concepts in the model, e.g., explore mediation effects between Self-Efficacy, Dominance, and Flow. A comparative study may directly test the effects of the Avatar.

## 9 Conclusion and future work

This article described a model and a method for the configuration of an industrial collaborative robot (cobot) with social interaction abilities thanks to its embodiment in an interactive virtual agent (avatar). Combined in a covatar, they can support workers in emotion co-regulation and Flow experiences during collaboration. The systematic conceptualisation of the BASSF model and its evaluation adds to the growing body of research on the use of technology to support emotion co-regulation in the workplace.

The results provide valuable insights into the assumptions underlying the BASSF Model, the real-time continuous emotional modeling method, and the aligned behavioral model to support emotion regulation through co-regulation agents. The article introduces a post-processing technique that enables reliable utilization of Pleasure, Arousal, and Dominance (PAD) social signals to activate BASSF-driver interventions from a covatar.

On the whole, future studies may test the difference between subjective user experiences and objective measures, and identify reasons of any discrepancies, and evaluate these effects in the long-term. Insights from such an approach, can point to which improvements are necessary to the BASSF model, as they do not serve the goal of the system to increase user-wellbeing long term. This is different from improvements at the perceptual level that need to be treated, for instance, by changing how the BASSF model is communicated at the surface level. Future work may also explore the effect of more complex and demanding work task and employ co-design approaches to tailor the approach to the needs of the workers, and their employers.

## Data Availability

The raw data supporting the conclusions of this article will be made available by the authors, without undue reservation.

## Footnotes

1 To protect the privacy of the participants, only gender-neutral pronouns will be used.

2 Multiple words, fillers, and incomplete sentences have been removed from all the participant’s citations.

## References

[B1] Abuhamdeh S. (2021). “On the relationship between flow and enjoyment,” in Advances in flow research. Editors Peifer C. Engeser S. (Cham: Springer International Publishing), 155–169. 10.1007/978-3-030-53468-4_6

[B2] Alves da Silva C. Hilpert B. Bhuvaneshwara C. Gebhard P. Nunnari F. Tsovaltzi D. (2023). “Visual similarity for socially interactive agents that support self-awareness,” in Proceedings of the 23rd ACM international conference on intelligent virtual agents, 1–3.

[B3] Arai T. Kato R. Fujita M. (2010). Assessment of operator stress induced by robot collaboration in assembly. CIRP Ann. 59, 5–8. 10.1016/j.cirp.2010.03.043

[B4] Arora R. Nicora M. L. Prajod P. Panzeri D. André E. Gebhard P. (2022). Employing socially interactive agents for robotic neurorehabilitation training 10.48550/ARXIV.2206.01587 Publisher: arXiv Version Number: 1 PMC11634856 39668889

[B5] Aymerich-Franch L. Petit D. Ganesh G. Kheddar A. (2017). Object touch by a humanoid robot avatar induces haptic sensation in the real hand. J. Computer-Mediated Commun. 22, 215–230. Cited By 12. 10.1111/jcc4.12188

[B6] Bakker I. van der Voordt T. Vink P. de Boon J. (2014). Pleasure, arousal, dominance: mehrabian and Russell revisited. Curr. Psychol. 33, 405–421. 10.1007/s12144-014-9219-4

[B7] Bambrah V. Moynihan A. B. Eastwood J. D. (2023). Self-focused but lacking self-knowledge: the relation between boredom and self-perception. J. Boredom Stud. 1.

[B8] Bartholomeyczik K. Knierim M. T. Weinhardt C. (2023). Fostering flow experiences at work: a framework and research agenda for developing flow interventions. Front. Psychol. 14, 1143654. 10.3389/fpsyg.2023.1143654 37484110 PMC10360049

[B9] Bazarevsky V. Kartynnik Y. Vakunov A. Raveendran K. Grundmann M. (2019). Blazeface: sub-millisecond neural face detection on mobile gpus. *arXiv preprint arXiv:1907.05047*

[B10] Beyrodt S. Nicora M. L. Nunnari F. Chehayeb L. Prajod P. Simeonovski T. (2023). “Socially interactive agents as cobot avatars: developing a model to support flow experiences and weil-being in the workplace,” in Proceedings of the 23rd ACM International Conference on Intelligent Virtual Agents (New York, NY: Association for Computing Machinery), 8.

[B11] Brambilla C. Malosio M. Reni G. Scano A. (2022). Optimal biomechanical performance in upper-limb gestures depends on velocity and carried load. Biology 11, 391. 10.3390/biology11030391 35336765 PMC8945111

[B12] Cao J. Wang H. Hu P. Miao J. (2008). “PAD model based facial expression analysis,” in Advances in visual computing. Editors Bebis G. Boyle R. Parvin B. Koracin D. Remagnino P. Porikli F. (Berlin, Heidelberg: Springer: Lecture Notes in Computer Science), 450–459. 10.1007/978-3-540-89646-3_44

[B13] Chehayeb L. Tsovaltzi D. Arora R. Gebhard P. (2021). “Individual differences and the function of emotions in socio-emotional and cognitive conflict: if an agent shames you, will you still be bored?,” in *2021 9th international Conference on affective Computing and intelligent interaction Workshops and demos (ACIIW)* (nara, Japan: IEEE), 1–8. 10.1109/ACIIW52867.2021.9666343

[B14] Csikszentmihalyi M. (1975). Beyond boredom and anxiety. Jossey-Bass behavioral science series (Jossey-Bass Publishers.

[B15] Csikszentmihalyi M. (1990). Flow: the psychology of optimal experience. 1st edn. New York: Harper and Row.

[B16] Csikszentmihalyi M. Larson R. (1987). Validity and reliability of the experience-sampling method. J. Nerv. Ment. Dis. 175, 526–536. 10.1097/00005053-198709000-00004 3655778

[B17] D’Agostino R. (1971). An omnibus test of normality for moderate and large sample sizes. Biometrika 58, 1–348. 10.1093/biomet/58.2.341

[B18] D’Agostino R. Pearson E. S. (1973). Tests for departure from normality. Empirical results for the distributions of b 2 and √b 1. Biometrika 60, 613–622. 10.2307/2335012

[B19] Dai L. Broekens J. Truong K. P. (2019). “Real-time pain detection in facial expressions for health robotics,” in 2019 8th international conference on affective computing and intelligent interaction workshops and demos (ACIIW) (Cambridge, United Kingdom: IEEE), 277–283. 10.1109/ACIIW.2019.8925192

[B20] Damiano R. Gena C. Lombardo V. Nunnari F. Pizzo A. (2008). A stroll with carletto: adaptation in drama-based tours with virtual characters. User Model. User-Adapted Interact. 18, 417–453. 10.1007/s11257-008-9053-1

[B21] [Dataset] Nicora M. L. Beyrodt S. Tsovaltzi D. Nunnari F. Gebhard P. Malosio M. (2023). Towards social embodied cobots: the integration of an industrial cobot with a social virtual agent

[B22] Detry M. A. Ma Y. (2016). Analyzing repeated measurements using mixed models. JAMA 315, 407. 10.1001/jama.2015.19394 26813213

[B23] D’Mello S. Graesser A. (2013). Autotutor and affective autotutor: learning by talking with cognitively and emotionally intelligent computers that talk back. ACM Trans. Interact. Intell. Syst. 2, 1–39. 10.1145/2395123.2395128

[B24] Elison J. Lennon R. Pulos S. (2006). Investigating the compass of shame: the development of the compass of shame scale. Soc. Behav. Personality Int. J. 34, 221–238. 10.2224/sbp.2006.34.3.221

[B25] Enders C. K. Tofighi D. (2007). Centering predictor variables in cross-sectional multilevel models: a new look at an old issue. Psychol. Methods 12, 121–138. 10.1037/1082-989X.12.2.121 17563168

[B26] Engeser S. (2012). Advances in flow research (New York, NY: Springer). 10.1007/978-1-4614-2359-1

[B27] Engeser S. Schiepe-Tiska A. Peifer C. (2021). “Historical lines and an overview of current research on flow,” in Advances in flow research. Editors Peifer C. Engeser S. (Cham: Springer International Publishing), 1–29. 10.1007/978-3-030-53468-4_1

[B28] Feuchter M. D. Preckel F. (2022). Reducing boredom in gifted education—evaluating the effects of full-time ability grouping. J. Educ. Psychol. 114, 1477–1493. 10.1037/edu0000694

[B29] Fisher C. D. (1987). “Boredom: construct, causes and consequences,”. Texas a and m univ college station dept of management. Section: Technical Reports.

[B30] Fonagy P. Gergely G. Jurist E. L. (2018). Affect regulation, mentalization and the development of the self. London, England: Routledge.

[B31] Fonagy P. Gergely G. Target M. (2007). The parent–infant dyad and the construction of the subjective self. J. child Psychol. psychiatry 48, 288–328. 10.1111/j.1469-7610.2007.01727.x 17355400

[B32] Fullagar C. J. Kelloway E. K. (2009). Flow at work: an experience sampling approach. J. Occup. Organ. Psychol. 82, 595–615. 10.1348/096317908X357903

[B33] Gebhard P. Mehlmann G. Kipp M. (2012a). Visual SceneMaker-a tool for authoring interactive virtual characters. J. Multimodal User Interfaces 6, 3–11. 10.1007/s12193-011-0077-1

[B34] Gebhard P. Mehlmann G. Kipp M. (2012b). Visual SceneMaker—a tool for authoring interactive virtual characters. J. Multimodal User Interfaces 6, 3–11. 10.1007/s12193-011-0077-1

[B35] Gebhard P. Schneeberger T. Dietz M. André E. Bajwa N. U. H. (2019). “Designing a mobile social and vocational reintegration assistant for burn-out outpatient treatment,” in *Proceedings of the 19th ACM international Conference on intelligent virtual agents* (paris France: ACM), 13–15. 10.1145/3308532.3329460

[B36] Gergely G. (2004). The role of contingency detection in early affect-regulative interactions and in the development of different types of infant attachment. Soc. Dev. 13, 468–478. 10.1111/j.1467-9507.2004.00277.x

[B37] Gilroy S. W. Cavazza M. Benayoun M. (2009). “Using affective trajectories to describe states of flow in interactive art,” in Proceedings of the international conference on advances in computer enterntainment technology (New York, NY, USA: Association for Computing Machinery), 165–172. ACE ’09. 10.1145/1690388.1690416

[B38] Heimbuch S. Bodemer D. (2017). “Effects of implicit guidance on contribution quality in a wiki-based learning environment,” in International conference of the learning sciences.

[B39] Järvelä S. Järvenoja H. Malmberg J. (2019). Capturing the dynamic and cyclical nature of regulation: methodological Progress in understanding socially shared regulation in learning. Int. J. Computer-Supported Collab. Learn. 14, 425–441. 10.1007/s11412-019-09313-2

[B40] Kato R. Fujita M. Arai T. (2010). “Development of advanced cellular manufacturing system with human-robot collaboration,” in 19th international symposium in robot and human interactive communication (IEEE), 355–360.

[B41] Kawamichi H. Kikuchi Y. Ueno S. (2005). Magnetoencephalographic measurement during two types of mental rotations of three-dimensional objects. IEEE Trans. Magnetics 41, 4200–4202. 10.1109/TMAG.2005.854802

[B42] Keyes C. L. M. (2002). The mental health Continuum: from languishing to flourishing in life. J. Health Soc. Behav. 43, 207. 10.2307/3090197 12096700

[B43] Keyes C. L. M. (2005). Mental illness and/or mental health? Investigating axioms of the complete state model of health. J. Consult. Clin. Psychol. 73, 539–548. 10.1037/0022-006X.73.3.539 15982151

[B44] Knudsen M. Kaivo-Oja J. (2020). Collaborative robots: frontiers of current literature. J. Intelligent Syst. Theory Appl. 3, 13–20. 10.38016/jista.682479

[B45] Kruskal W. H. Wallis W. A. (1952). Use of ranks in one-criterion variance analysis. J. Am. Stat. Assoc. 47, 583–621. Publisher: Taylor and Francis _eprint. 10.1080/01621459.1952.10483441

[B46] Kurz M. Brüggemeier B. Breiter M. (2021). “Success is not final; failure is not fatal – task success and user experience in interactions with Alexa, Google assistant and Siri,”. Human-computer interaction. Design and user experience case studies. Editor Kurosu M. (Cham: Springer International Publishing), 12764, 351–369. 10.1007/978-3-030-78468-3_24

[B47] Lackas J. (2021). Explicit and implicit guidance to emotion regulation to support collaborative tasks: a model based on socio-cognitive conflict and flow parameters. Ph.D. thesis, Univ. Des. Saarl.

[B48] Loksa D. Ko A. J. Jernigan W. Oleson A. Mendez C. J. Burnett M. M. (2016). “Programming, problem solving, and self-awareness: effects of explicit guidance,” in Proceedings of the 2016 CHI conference on human factors in computing systems (New York, NY, USA: Association for Computing Machinery), 16, 1449–1461. 10.1145/2858036.2858252

[B49] Lugrin B. Pelachaud C. Traum D. (2022). The handbook on socially interactive agents: 20 Years of research on embodied conversational agents, intelligent virtual agents, and social robotics volume 2: interactivity, platforms, application. 48 (New York, NY, USA: Association for Computing Machinery).

[B50] Massimini F. Csikszentmihalyi M. Carli M. (1987). The monitoring of optimal experience A tool for psychiatric rehabilitation. J. Nerv. Ment. Dis. 175, 545–549. 10.1097/00005053-198709000-00006 3655780

[B51] Matthews R. A. Pineault L. Hong Y.-H. (2022). Normalizing the use of single-item measures: validation of the single-item compendium for organizational psychology. J. Bus. Psychol. 37, 639–673. 10.1007/s10869-022-09813-3

[B52] Mavridis N. (2015). A review of verbal and non-verbal human-robot interactive communication. Robotics Aut. Syst. 63, 22–35. 10.1016/j.robot.2014.09.031

[B53] Mayring P. (2014). Qualitative content analysis, 145.

[B54] McRae K. Gross J. J. (2020). Emotion regulation. Emotion 20, 1–9. Publisher: US: American Psychological Association. 10.1037/emo0000703 31961170

[B55] Mehrabian A. (1995). Framework for a comprehensive description and measurement of emotional states. Genet. Soc. general Psychol. Monogr. 121, 339–361. 7557355

[B56] Mehrabian A. (1996a). Analysis of the big-five personality factors in terms of the pad temperament model. Aust. J. Psychol. 48, 86–92. 10.1080/00049539608259510

[B57] Mehrabian A. (1996b). Pleasure-arousal-dominance: a general framework for describing and measuring individual differences in Temperament. Curr. Psychol. 14, 261–292. 10.1007/BF02686918

[B58] Mehrabian A. Russell J. A. (1974). An approach to environmental psychology/Albert Mehrabian and James A. Russell: MIT Press.

[B59] Mollahosseini A. Hassani B. Mahoor M. H. (2019). Affectnet: a database for facial expression, valence, and arousal computing in the wild. IEEE Trans. Affect. Comput. 10, 18–31. 10.1109/TAFFC.2017.2740923

[B60] Mondellini M. Prajod P. Lavit Nicora M. Chiappini M. Micheletti E. Storm F. A. (2023). Behavioral patterns in robotic collaborative assembly: comparing neurotypical and Autism Spectrum Disorder participants. Front. Psychol. 14, 1245857. 10.3389/fpsyg.2023.1245857 37954185 PMC10637657

[B61] Moneta G. B. (2021). On the conceptualization and measurement of flow. Cham: Springer International Publishing, 31–69. 10.1007/978-3-030-53468-4_2

[B62] Nathanson D. L. (1994). Shame and pride: affect, sex, and the birth of the self. WW Norton and Company.

[B63] Nicora M. L. André E. Berkmans D. Carissoli C. D’Orazio T. Delle Fave A. (2021). “A human-driven control architecture for promoting good mental health in collaborative robot scenarios,” in 2021 30th IEEE international conference on robot and human interactive communication (RO-MAN) (IEEE), 285–291.

[B64] Nomura T. Kanda T. Suzuki T. (2006). Experimental investigation into influence of negative attitudes toward robots on human–robot interaction. AI and Soc. 20, 138–150. 10.1007/s00146-005-0012-7

[B65] Nunnari F. Heloir A. (2019). Yet another low-level agent handler. Comput. Animat. Virtual Worlds 30, e1891. 10.1002/cav.1891

[B66] Nunnari F. Nicora M. L. Prajod P. Beyrodt S. Chehayeb L. Andre E. (2023). “Understanding and mapping pleasure, arousal and dominance social signals to robot-avatar behavior,” in 2023 11th international conference on affective computing and intelligent interaction workshops and demos (ACIIW) (Cambridge, MA, USA: IEEE), 1–8. 10.1109/ACIIW59127.2023.10388078

[B67] Ojstersek R. Buchmeister B. Javernik A. (2023). The importance of cobot speed and acceleration on the manufacturing system efficiency. Procedia Comput. Sci. 217, 147–154. 10.1016/j.procs.2022.12.210

[B68] Pagalyte E. Mancini M. Climent L. (2020). “Go with the flow: Reinforcement learning in turn-based battle video games,” in Proceedings of the 20th ACM international conference on intelligent virtual agents (New York, NY, USA: Association for Computing Machinery). IVA ’20. 10.1145/3383652.3423868

[B69] Prajod P. Huber T. André E. (2022a). “Using explainable ai to identify differences between clinical and experimental pain detection models based on facial expressions,” in MultiMedia modeling: 28th international conference, MMM 2022, Phu Quoc, Vietnam, June 6–10, 2022, Proceedings, Part I (Springer), 311–322.

[B70] Prajod P. Schiller D. Huber T. André E. (2022b). “Do deep neural networks forget facial action units? exploring the effects of transfer learning in health related facial expression recognition,” in AI for disease surveillance and pandemic intelligence: intelligent disease detection in action (Springer), 217–233.

[B71] Quigley M. Conley K. Gerkey B. Faust J. Foote T. Leibs J. (2009). Ros: an open-source robot operating system. ICRA workshop open source Softw. Kobe, Jpn. 3, 5.

[B72] Rheinberg F. Vollmeyer R. Engeser S. (2003). “Flow short scale,” in Diagnostik von Selbstkonzept, Lernmotivation und Selbstregulation. *[Diagnosis of Motivation and Self-Concept]* (Göttingen: Hogrefe).

[B73] Russakovsky O. Deng J. Su H. Krause J. Satheesh S. Ma S. (2015). Imagenet large scale visual recognition challenge. Int. J. Comput. Vis. 115, 211–252. 10.1007/s11263-015-0816-y

[B74] Russell J. A. Mehrabian A. (1977). Evidence for a three-factor theory of emotions. J. Res. Personality 11, 273–294. 10.1016/0092-6566(77)90037-X

[B75] Samrose S. Anbarasu K. Joshi A. Mishra T. (2020). “Mitigating boredom using an empathetic conversational agent,” in Proceedings of the 20th ACM international conference on intelligent virtual agents (New York, NY, USA: Association for Computing Machinery). IVA ’20. 10.1145/3383652.3423905

[B76] Schneiders E. Papachristos E. (2022). “It’s not all bad-worker perceptions of industrial robots,” in 2022 17th ACM/IEEE international conference on human-robot interaction (HRI) (IEEE), 1025–1029.

[B77] Simonyan K. Zisserman A. (2014). Very deep convolutional networks for large-scale image recognition. arXiv Prepr. arXiv:1409, 1556.

[B78] Toisoul A. Kossaifi J. Bulat A. Tzimiropoulos G. Pantic M. (2021a). Estimation of continuous valence and arousal levels from faces in naturalistic conditions. Nat. Mach. Intell. 3, 42–50. 10.1038/s42256-020-00280-0

[B79] Toisoul A. Kossaifi J. Bulat A. Tzimiropoulos G. Pantic M. (2021b). Estimation of continuous valence and arousal levels from faces in naturalistic conditions. Nat. Mach. Intell. 3, 42–50. 10.1038/s42256-020-00280-0

[B80] Tze V. Daniels L. Klassen R. (2014). Examining the factor structure and validity of the English precursors to boredom scales. Learn. Individ. Differ. 32, 254–260. 10.1016/j.lindif.2014.03.018

[B81] Wagner J. Lingenfelser F. Baur T. Damian I. Kistler F. André E. (2013). “The social signal interpretation (ssi) framework: multimodal signal processing and recognition in real-time,” in Proceedings of the 21st ACM international conference on Multimedia, 831–834.

[B82] Xu X. Lu Y. Vogel-Heuser B. Wang L. (2021). Industry 4.0 and industry 5.0—inception, conception and perception. J. Manuf. Syst. 61, 530–535. 10.1016/j.jmsy.2021.10.006

